# Proteomic discovery of MNT as a novel interacting partner of E3 ubiquitin ligase E6AP and a key mediator of myeloid differentiation

**DOI:** 10.18632/oncotarget.6156

**Published:** 2015-10-25

**Authors:** Isha Kapoor, Jitendra Kanaujiya, Yogesh Kumar, Jagadeshwar Reddy Thota, Madan L.B. Bhatt, Naibedya Chattopadhyay, Sabyasachi Sanyal, Arun Kumar Trivedi

**Affiliations:** ^1^ Biochemistry Division, CSIR-Central Drug Research Institute, Lucknow, UP, India; ^2^ Department of Radiotherapy, King George's Medical University, Lucknow, UP, India; ^3^ SAIF Division, CSIR-Central Drug Research Institute, Lucknow, UP, India; ^4^ Division of Endocrinology and Center for Research in Anabolic Skeletal Targets in Health and Illness (ASTHI), CSIR-Central Drug Research Institute (CSIR-CDRI), Lucknow, UP, India

**Keywords:** MNT, E6AP, ATRA, mass spectrometry, APL

## Abstract

Perturbed stability of regulatory proteins is a major cause of transformations leading to cancer, including several leukemia subtypes. Here, for the first time we demonstrate that E6-associated protein (E6AP), an E3 ubiquitin ligase negatively targets MAX binding protein MNT for ubiquitin-mediated proteasome degradation and impedes ATRA mediated myeloid cell differentiation. MNT is a member of the Myc/Max/Mad network of transcription factor that regulates cell proliferation, differentiation, cellular transformation and tumorigenesis. Wild-type E6AP promoted proteasome dependent degradation of MNT, while catalytically inactive E6AP having cysteine replaced with alanine at amino-acid 843 position (E6APC843A) rather stabilized it. Further, these proteins physically associated with each other both in non-myeloid (HEK293T) and myeloid cells. MNT overexpression induced G0-G1 growth arrest and promoted myeloid differentiation while its knockdown mitigated even ATRA induced differentiation suggesting MNT to be crucial for myeloid differentiation. We further showed that ATRA inhibited E6AP and stabilized MNT expression by protecting it from E6AP mediated ubiquitin-proteasome degradation. Notably, E6AP knockdown in HL60 cells restored MNT expression and promoted myeloid differentiation. Taken together, our data demonstrated that E6AP negatively regulates granulocytic differentiation by targeting MNT for degradation which is required for growth arrest and subsequent myeloid differentiation by various differentiation inducing agents.

## INTRODUCTION

Myeloid leukemia represents a heterogenous malignancy of hematopoietic stem cell disorders characterized by differentiation blockade and over-proliferation of clonal myeloid cells, resulting in accumulation of non-functional myeloid cells, termed myeloblasts [1-4]. This differentiation blockade of myeloblasts may be overcome by forcing them to differentiate by differentiation inducing agents such as All *-trans* retinoic acid (ATRA), Vitamin D_3_ or PMA. ATRA is the prototype for the cancer differentiation therapy in APL used either alone or in rational combination with other chemotherapeutic agents. The use of ATRA with chemotherapy was a major breakthrough in the treatment of APL, with complete remission in about 90% patients. The biological effects of ATRA are mediated through nuclear receptors; retinoic acid receptors (RARs) and retinoid X receptor (RXR) which bind to retinoic acid response elements (RAREs) [5, 6]. However, the underlying ATRA targets and downstream signalling involved in growth arrest and induction of differentiation are yet to be identified. In recent years, perturbed stability of regulatory proteins due to dysregulation of E3 ubiquitin ligases has emerged as a major cause of transformation leading to cancer, including several leukemia subtypes [7, 8]. These E3 ligases are unique in the sense that they provide substrate specificity as to which protein is subjected to ubiquitin-mediated proteasome degradation.

Ubiquitin-protein ligase E6-associated protein (E6AP; a 100kDa cellular protein), founding member of the HECT (homologous with E6AP C terminus) family proteins is one such E3 ubiquitin ligase implicated in the degradation of the tumour suppressor TP53 [9] and other cell-cycle regulatory proteins [10]. Deregulation of the E3 activity of E6AP has been associated with the development of human diseases such as cervical carcinogenesis, Angelman syndrome and others [11]. In fact, in a previous study using mass spectrometry based proteomics approach we have also identified ubiquitin-protein ligase E6AP as a target of tamoxifen in MCF7 breast cancer cells [12]. In a previous study, we demonstrated that ubiquitin-protein ligase E6AP may negatively regulate granulopoiesis by targeting tumour suppressor C/EBPα for ubiquitin-mediated proteasomal degradation [13]. Moreover, there are several reports that indicate ubiquitin-mediated degradation of short-lived regulatory proteins including cell-cycle regulatory proteins is essential for ATRA-mediated cellular functions [14, 15]. ATRA-induced myeloid differentiation of leukemia cells is accompanied by G0-G1 arrest, yet how ATRA couples cell-cycle arrest to differentiation therapy remains largely elusive. Unravelling this process may lead to more efficacious therapies for leukemia and other types of cancers. This prompted us to identify other putative substrates of ubiquitin-protein ligase E6AP from myeloid leukemia cells treated with ATRA. With this notion, we performed GST-pull down using GST-E6AP from lysates of ATRA induced HL60 cells and identified novel interacting partners of ubiquitin-protein ligase E6AP by proteomics based mass spectrometry.

Here, we identified MAX-binding protein MNT (also known as ROX, hereafter referred just as MNT) as a novel interacting partner of E6AP. MNT (74kDa), a nuclear protein is the member of the Myc/Max/Mad network of transcription factors that regulates cell proliferation, differentiation and cellular transformation. Similar to other proteins of the network, MNT heterodimerizes with Protein max and binds the canonical CACGTG E-box elements and regulates cell-cycle entry and promotes cellular differentiation [16]. Hurlin and co-workers showed MNT as a MAX-interacting transcriptional repressor and demonstrated that deletion of MNT leads to disrupted cell-cycle control and tumorigenesis [17]. Consistent with MNT functioning as a tumour suppressor, conditional inactivation of MNT in breast epithelium led to adenocarcinomas [17]. Nilsson and co-workers revealed MNT as a putative MYC antagonist and interestingly amassed substantial evidence to demonstrate that MNT loss triggers MYC transcription targets, proliferation, apoptosis and transformation [18]. Henceforth, substantial evidences demonstrate MNT as a putative MYC antagonist, and a potent transcriptional repressor.

Thus, in the present study we sought to identify novel interacting proteins of ubiquitin-protein ligase E6AP through mass spectrometry and further elucidated its significance in the pathophysiology of myeloid leukemia, wherein differentiation blockade is a conspicuous feature. Our study uncovers a novel finding demonstrating MNT as a novel substrate and interacting partner of ubiquitin protein ligase E6AP in non-myeloid and myeloid cells. In a nutshell, our data demonstrates MNT as a key mediator of ATRA induced myeloid growth arrest and granulocytic differentiation wherein ATRA rescues MNT from ubiquitin-mediated proteasome degradation by inhibiting ubiquitin-protein ligase E6AP.

## RESULTS

### MNT is a novel interacting protein of E6AP

**Figure 1:**
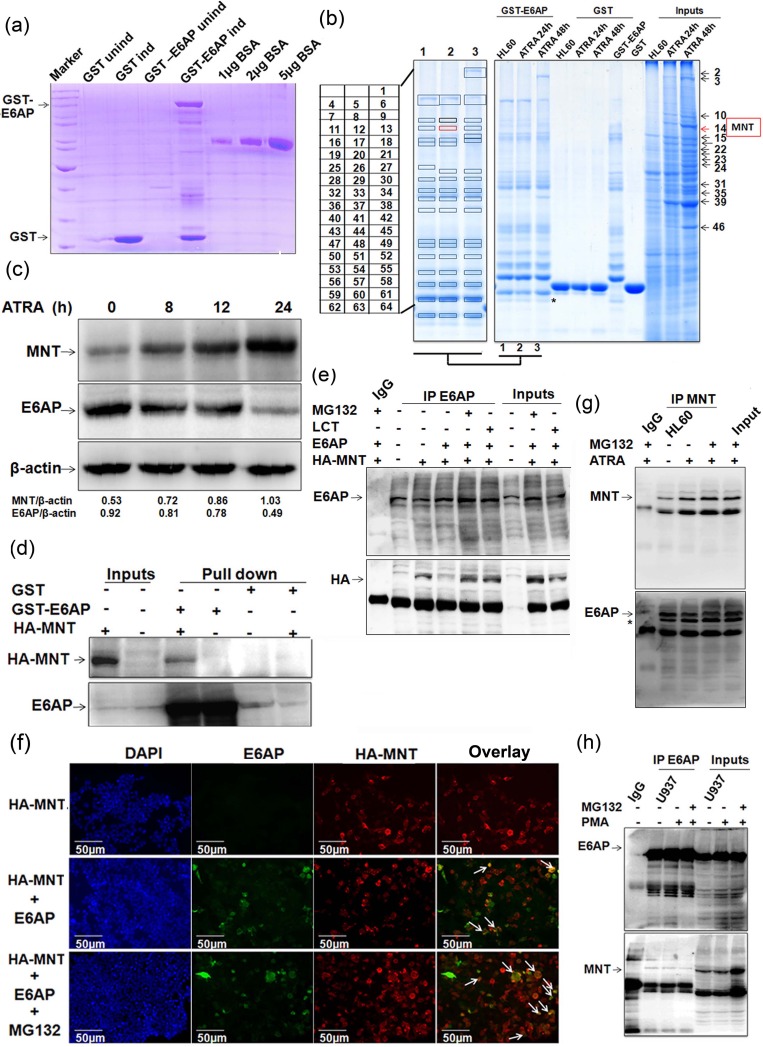
MNT as a novel interacting partner of E6AP **a.** GST and GST-E6AP proteins were expressed in *E. coli* with 0.2mM IPTG induction for 4h at 37°C and 16h at 20°C respectively, isolated and purified with glutathione sepharose 4B beads **b.** Colloidal coomassie stained gel showing GST pull down performed using GST and GST-E6AP from WCEs of 1.0μM ATRA treated HL60 cells. **c.** WCEs of 1.0μM ATRA treated HL60 cells for 0, 8, 12 and 24h were resolved on 8% SDS-PAGE and immunoblotted with MNT, E6AP and β-actin antibodies. **d.** GST pull-down with GST and GST-E6AP from lysates of 293T transfected with 0.5ug HA-MNT was performed. **e.** Co-immunoprecipitation with E6AP antibody was performed from lysates of HEK293T co-transfected with 1.0μg HA-MNT and 0.5μg E6AP as indicated. The blot was probed with anti-E6AP followed by anti-HA antibodies. Cells were treated with 10μM MG132 and 10μM Lactacystin (LCT) 6h prior to harvesting, as indicated. **f.** 293T cells were co-transfected with 0.5μg HA-MNT and 0.5μg E6AP. IFC was performed 24h post-transfection in order to avoid heavier degradation of MNT. We followed a regular IFC protocol and incubated cells overnight with ant-E6AP and anti-HA antibodies. Next day, cells were incubated with anti-rabbit (Alexafluor 594) and anti-mouse (Alexafluor 488) secondary antibodies. Cells were dried and mounted to capture the images; 10μM MG132 treatment was given 6h prior to fixing for immunostaining. **g.** HL60 cells were treated with 1μM ATRA for 24h and cells were treated with 10μM MG132 6h prior to harvesting as indicated. Co-immunoprecipitation using MNT antibody was performed and immunoblotted with anti-MNT followed by anti-E6AP antibody after stripping the same blot; * indicates unstripped MNT from upper panel. **h.** Endogenous E6AP was co-immunoprecipitated using E6AP antibody from U937 WCEs treated with 5nM PMA for 24h. Co-immunoprecipitates were resolved and probed with E6AP followed by MNT antibody. Cells were treated with 10μM MG132 6h prior to harvesting as indicated. Results are representative of minimum three independent experiments.

To identify ubiquitin-protein ligase E6AP interacting proteins, we performed *in vitro* GST-pull down using GST-tagged E6AP from whole cell extracts (WCEs) of HL60 cells. GST and GST-E6AP expressed in BL21 strain of *E.coli* were purified, resolved on 10% SDS-PAGE and visualized by coomassie staining along with different amounts of BSA so as to semi-quantitate the amounts of fusion protein expressed (Figure 1a). Purified fusion proteins were incubated with WCEs of HL60 cells treated with ATRA for 0, 24 and 48h, resolved on 10% SDS-PAGE and visualized by colloidal coomassie stain (Figure 1b). Ubiquitin-protein ligase E6AP-interacting proteins from ATRA treated and untreated HL60 cells as well as proteins showing robust changes in their expression upon ATRA induction (48h) from input lane were excised, digested with trypsin and subjected to MS/MS analysis using MALDI-TOF/TOF. In order to select the putative interacting proteins (Table S1) and to further explore their relevance in myeloid cell biology, we performed literature search and selected MAX-binding protein MNT, a transcriptional repressor [18] as a potential candidate; Notably, MNT was identified both as a protein interacting with E6AP and induced upon ATRA treatment. Peptide peaks and ion score of MNT peptides are mentioned in Supplementary Details (Figure S9). Immunoblotting ATRA treated HL60 WCEs with anti-MNT and anti-E6AP antibodies showed increase in MNT levels with concomitant decrease in E6AP levels (Figure 1c). We also assessed endogenous levels of MNT in various cell lines representing different myeloid leukemia subtypes. Immunoblotting with anti-MNT antibody showed MNT to be expressed in these AML cell lines (Figure S1a); however, its expression was lower than in PBMCs isolated from normal healthy volunteers. Moreover, a study by Guo et al, showing mutations in myc antagonists including MNT (Rox) in some of acute leuekmias also indicated a role of MNT in cell-cycle regulation and differentiation in myeloid cells [19]. Further, in order to verify the physical association of MNT with ubiquitin-protein ligase E6AP, we performed GST-pull down from lysates of HEK293T cells transfected with indicated expression plasmids. Immunoblotting confirmed physical interaction of E6AP and MNT thus verifying our proteomics data (Figure 1d). To assess physical interaction within cell, E6AP was co-immunoprecipitated from WCEs of 293T co-transfected with indicated plasmids. Immunoblotting with anti-HA followed by anti-E6AP antibody confirmed interaction between E6AP and HA-MNT (Figure 1e). Interestingly, in conditions treated with proteasome inhibitors (MG132 and Lactacystin), a prominent interaction between MNT and E6AP was observed (Figure 1e); obviously because inhibition of the proteasome pathway restored MNT. Since these two proteins physically associated with each other, we further assessed their co-localisation by co-transfecting HA-MNT and E6AP in 293T cells. Subsequent probing with anti-HA and anti-E6AP antibodies followed by incubation with alexafluor-594 and alexafluor-488 tagged secondary antibodies confirmed co-localisation of these proteins in the nuclear periphery (Figure 1f); intense co-localisation observed in MG132 treated condition is again in agreement with strong physical interaction observed in GST-pull down and co-immunoprecipitation assays (Figure 1e). Taken together, these data validated our proteomic findings and confirmed that E6AP and MNT physically associated with each other. In order to further substantiate their physical interaction in physiologically relevant myeloid cells, we assessed physical interaction between endogenous E6AP and MNT in myeloid leukemia cell line HL60. MNT was co-immunoprecipitated using anti-MNT antibody from HL60 WCEs and resolved on 8% SDS-PAGE. Immunoblotting with MNT and E6AP antibodies clearly showed physical interaction between the two proteins (Figure 1g). Expectedly, prominent physical interaction was observed yet again in MG132 treated conditions. Cell-free physical interaction of these two proteins was also confirmed in yet another myeloid cell line U937 cells (Figure 1h). Taken together, these data indicated that ubiquitin-protein ligase E6AP and MAX-binding protein MNT physically associated with each other both *in vitro* and in cell-free system.

### E6AP targets MNT for ubiquitin-mediated proteasome degradation

**Figure 2:**
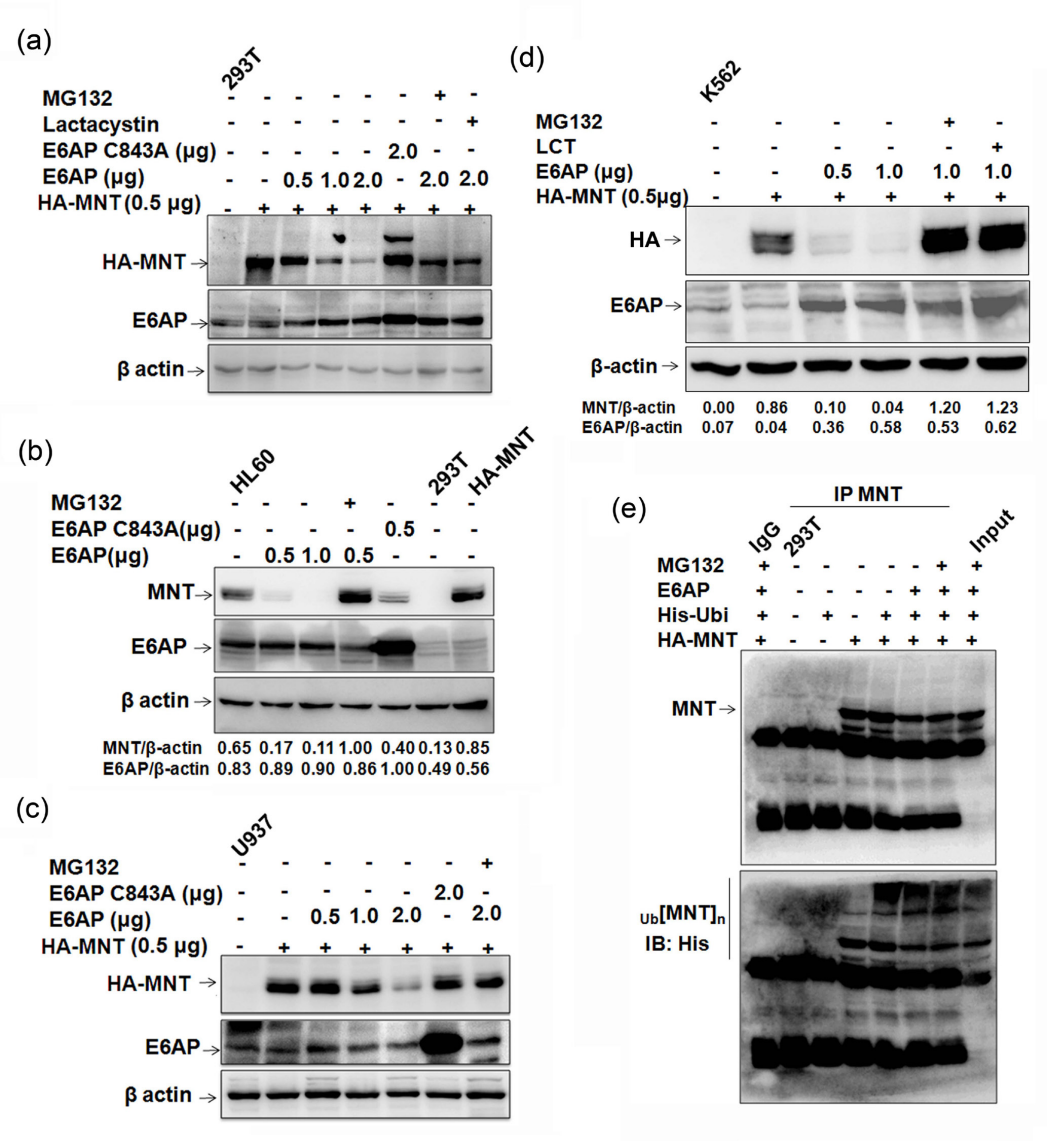
E6AP inhibits MNT steady state levels **a.** 293T cells were transfected with HA-MNT (0.5μg), E6AP (0.5, 1.0, 2.0μg) and E6APC843A-HA (2.0μg). 24h post transfection, lysates were prepared and immunoblotted with HA, E6AP and β-actin antibodies. Cells were treated with 10μM MG132 and 10μM Lactacystin 6h prior to harvesting, as indicated. **b.** HL60 cells were transfected with E6AP (0.5, 1.0μg) and E6APC843A-HA (0.5μg) and treated with 10μM MG132 6h prior to lysate preparation as indicated. Lysates resolved on 10% SDS-PAGE were immunoblotted with anti-MNT, E6AP and β-actin antibodies. **c.** U937 cells were transfected with HA-MNT (0.5μg), E6AP (0.5, 1.0, 2.0μg) and E6AP C843A (2.0μg) and treated with 10μM MG132 6h prior to lysate preparation. Lysates were immunoblotted with anti-HA, E6AP and β-actin antibodies. **d.** K562 cells were co-transfected with HA-MNT (0.5μg), E6AP (0.5, 1.0μg) and treated with 10μM MG132 and Lactacystin 6h prior to lysate preparation as indicated. Lysates were resolved on 8% SDS-PAGE and probed with anti-HA, E6AP and β-actin antibodies. **e.** 293T cells were transfected with HA-MNT (1.0μg), His-ubi (1.0μg) and E6AP (2.0μg); cells were treated with treated with 10μM MG132 6h prior to lysate preparation. MNT was co-immunoprecipitated and probed with anti-MNT antibody (upper panel). The same membrane was stripped and probed with anti-His antibody (lower panel). Results are representative of minimum three independent experiments.

E6AP is an E3 ubiquitin ligase for several target proteins and promotes their degradation *via* proteasome-mediated pathway [9, 10, 13, 20]. Further, as these two proteins physically interacted and an enhanced MNT protein expression in HL60 cells upon ATRA treatment with concomitant decrease in E6AP expression was observed, we next investigated if E6AP modulated MNT stability by targeting it for degradation. In order to evaluate the effects of E6AP on MNT protein steady-state levels, we used HEK293T cells which have no detectable MNT. 293T cells were transfected with 0.5μg HA-MNT together with either increasing doses of E6AP (0.5, 1.0, 2.0μg) or with 2.0μg of E6AP-C843A as indicated. 24h post-transfection, immunoblotting with anti-E6AP and anti-HA antibody showed that E6AP drastically down regulated MNT while mutant E6AP-C843A rather stabilized (Figure 2a), apparently due to its dominant negative effect over endogenous E6AP. This data demonstrated that E6AP promoted the degradation of MNT protein. Further, treatment with MG132 and Lactacystin as indicated markedly inhibited MNT degradation suggesting this degradation to be mediated *via* proteasome pathway. In order to substantiate further, we assessed if E6AP could modulate endogenous MNT expression in physiological settings. Transfection of E6AP in HL60 cells also inhibited endogenous MNT expression while E6AP-C843A rather stabilized MNT (Figure 2b). MG132 treatment also restored endogenous MNT, suggesting E6AP targeted endogenous MNT for proteasome-mediated degradation in physiologically relevant myeloid cells. Similar results were observed in yet other myeloid cell lines U937 (Figure 2c) and K562 cells (Figure 2d). Because E6AP destabilized MNT apparently by targeting it for proteasome mediated degradation, we next determined if E6AP promoted its ubiquitination. To address this, we performed in-cell ubiquitination assay in 293T cells by transiently transfecting either HA-MNT alone or together with His-ubi and E6AP as indicated. Cells were treated with 10μM MG132 6h prior to harvesting as indicated. 24h post-transfection, MNT was co-immunoprecipitated and probed with anti-His and anti-MNT antibodies. Increased polyubiquitination of MNT (heavy ladder) in HA-MNT, E6AP and His-ubi transfected condition while more intense polyubiquitinated MNT was observed in cells treated with MG132 suggesting E6AP promoted MNT ubiquitination (upper panel Figure 2e). Slightly low polyubiquitinated MNT observed in HA-MNT, E6AP and His-ubi condition compared to HA-MNT and His-ubi transfected condition (upper panel) was obviously due to rapid degradation of ubiquitinated MNT through proteoasome pathway (Lower panel). Taken together, these data demonstrated that E6AP modulated MNT protein stability *via* promoting its degradation through ubiquitin-mediated proteasome pathway.

### MNT expression is upregulated during myeloid differentiation

**Figure 3:**
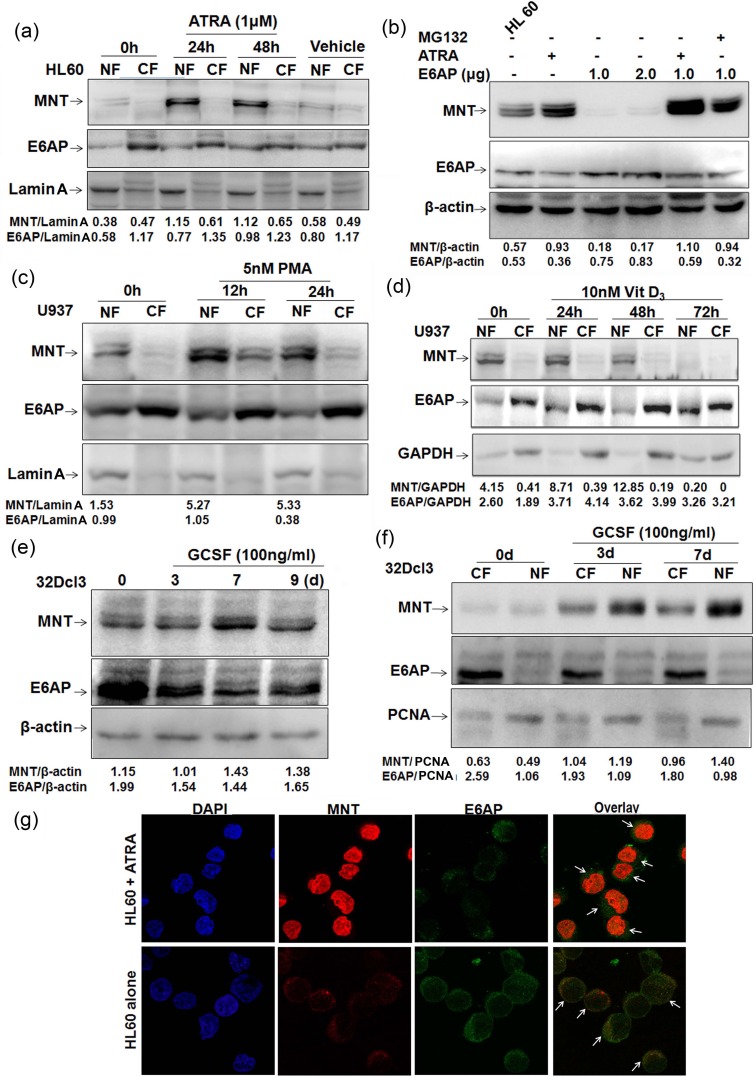
MNT expression is upregulated during myeloid differentiation **a.** HL60 cells were treated with 1μM ATRA. Post-ATRA treatment for 0, 24 and 48h lysates from nuclear and cytoplasmic fractions were prepared, resolved on 10% SDS-PAGE and immunoblotted with anti-MNT and anti-E6AP antibodies. Lamin A was used as loading control for the quality of nuclear fractions. DMSO was used as vehicle. **b.** HL60 cells were transfected with increasing amounts of E6AP (1.0 and 2.0μg), treated with 1μM ATRA for 24h in control cells and 24h post transfection for next 24h in 1.0μg E6AP transfected cells. As indicated 10μM MG132 treatment was given 6h prior to lysate preparation. Lysates resolved on 10% SDS-PAGE were immunoblotted with anti-MNT, E6AP and β-actin antibodies. **c.** U937 cells were treated with 5nM PMA. Post-PMA treatment for 0, 12 and 24h lysates from nuclear and cytoplasmic fractions were prepared, resolved on 10% SDS-PAGE and immunoblotted with anti-MNT followed by anti-E6AP antibodies. Lamin A was used as loading control. **d.** U937 cells were treated with 10nM Vitamin D_3_, post-Vitamin D_3_ treatment for 0, 24, 48 and 72h, lysates from nuclear and cytoplasmic fractions were prepared, resolved on 10% SDS-PAGE and immunoblotted with anti-MNT followed by anti-E6AP and anti-GAPDH antibodies. **e.** 32Dcl3 cells were treated with 100ng/ml G-CSF for 0, 3, 7 and 9 days as indicated. Post-induction, WCEs were prepared, resolved on 10% SDS-PAGE and immunoblotted with anti-MNT and anti-E6AP antibodies. β-actin was used as loading control. **f.** 32Dcl3 cells were treated with 100ng/ml G-CSF as indicated. Post-induction, nuclear/cytoplasmic fractions were prepared, resolved on 10% SDS-PAGE followed by immunoblotting with anti-MNT and anti-E6AP antibodies. PCNA was used a loading control for the quality of nuclear fractions. **g.** Indirect immunofluorescence staining for MNT and E6AP was performed using respective primary and conjugated secondary antibodies. Stained cells were visualized under fluorescence microscope (63X) to assess their localization. Results are representative of three minimum independent experiments.

Because DMSO, a known inducer of myeloid differentiation in HL60 cells has been shown to enhance MNT protein levels in HL60 cells [16] and moreover we also identified MNT from ATRA induced HL60 cells, we assumed ATRA might also enhance MNT protein levels. As MNT is a nuclear protein, we assessed MNT protein levels in nuclear/cytoplasmic fractions of HL60 cells treated with ATRA for 0, 24 and 48h. Immunoblotting with MNT antibody showed persistent increase in MNT protein levels in nuclear fractions, while interestingly; E6AP protein levels decreased in the nuclear fractions (Figure 3a). DMSO (>0.1%) as a vehicle showed no change. In order to determine if ATRA enhanced MNT protein levels by inhibiting E6AP in HL60 cells, we overexpressed E6AP in HL60 cells and treated them with ATRA. E6AP inhibited endogenous MNT levels while, ATRA treatment like MG132 strongly restored MNT even in E6AP overexpressed HL60 cells, suggesting ATRA enhanced MNT expression apparently by inhibiting E6AP-mediated ubiquitination of MNT (Figure 3b). In order to substantiate the apparent ability of ATRA inhibiting E6AP-mediated MNT ubiquitination, we performed ubiquitination assay in HL60 cells by transiently transfecting His-Ubi or E6AP as indicated. Cells were also treated with 1μM ATRA and MG132 as indicated. 24h post-transfection endogenous MNT was co-immunoprecipitated and probed with anti-His and anti-MNT antibodies. Increased polyubiquitination of MNT (heavy ladder) in E6AP and His-Ubi transfected condition (fourth lane lower panel) was observed while, interestingly, low polyubiquitinated MNT was observed in E6AP and His-Ubi condition treated with ATRA (fifth lane lower panel) (Figure S1b), suggesting ATRA indeed inhibited E6AP-mediated degradation of MNT (Lower panel). Therefore, it is very likely that these differentiation inducers might promote differentiation by inhibiting E6AP-mediated ubiquitination and subsequent degradation of MNT; albeit it still warrants detailed investigation to address if ATRA regulates E6AP ligase activity. Because like DMSO, ATRA also induced MNT protein levels, we therefore, asked if increase in MNT protein levels during differentiation is a general pre-requisite event. To this end, we treated another myeloid cell line U937 cells with 5nM PMA, a monocytic differentiation inducer [21] for 0, 12 and 24h. Post-PMA treatment, immunoblotting with MNT antibody clearly showed similar transient burst in MNT protein levels in nuclear fractions (Figure 3c). In contrast, E6AP showed reduced levels in nuclear fractions with concomitant increase in cytoplasmic fractions. Further, U937 cells were treated with another well-known myeloid differentiation inducer, 10nM Vitamin D_3_ [22]. Interestingly, Vitamin D_3_ treatment also induced MNT expression yet again in nuclear fractions, while E6AP showed enhanced levels in cytoplasmic fractions (Figure 3d). In light of above observations, we also investigated levels of MNT expression during a cytokine (G-CSF) induced myeloid differentiation. For this, we exploited the innate ability of a myeloblastic 32Dcl3 cells (derived from normal mouse bone marrow) to undergo terminal differentiation by G-CSF [23]. 32Dcl3 cells were treated with G-CSF as indicated. Post-G-CSF treatment, WCEs were prepared and resolved on 10% SDS-PAGE. Immunoblotting with anti-MNT antibody revealed increased levels of MNT 7-days post-G-CSF induction. Interestingly, E6AP protein levels yet again revealed inverse relation with MNT (Figure 3e). We also observed increased expression levels of MNT in nuclear fractions of 32Dcl3 cells post-3 and 7-days of G-CSF induction (Figure 3f) which yet again indicated that MNT upregulation is a general phenomenon during granulopoiesis. In agreement with these observations, we next assessed the localization of these two proteins by indirect immunofluorescence in HL60 cells. Interestingly, significantly increased expression of endogenous MNT with simultaneous decrease in E6AP was observed in the nucleus upon ATRA treatment unlike control cells (untreated HL60 cells); the overlay of the two images showed that both proteins co-localized in the nuclear and cytoplasmic interface (Figure 3g). Control cells exhibited dispersed expression of MNT both in the nucleus and cytoplasm while E6AP was more in the cytoplasm. These results demonstrated that the expression of nuclear MNT is transiently upregulated during myeloid differentiation with concomitant decrease in E6AP; suggesting E6AP inhibition is required for increased steady state levels of MNT during myeloid differentiation. Next, we investigated the localization of MNT and c-Myc by indirect immunofluorescence in HL60 cells since these proteins are antagonist to each other; c-Myc, an important regulator of cell cycle entry is strongly induced during the G0 to S-phase transition [24], while Myc-antagonist, MNT is upregulated in the nucleus during ATRA induced myeloid differentiation (Figure S1.c). Notably, formation of heterodimeric Mnt-Max complex regulates cell cycle entry and is essential for differentiation [24], we therefore, investigated the MNT-MAX complex formation during 1μM ATRA induced differentiation of HL60 cells and observed a strong Mnt-Max interaction upon ATRA induction (Figure S1d) Taken together, these data thus consolidated our hypothesis that enhanced MNT protein levels (and in turn increased Mnt-Max heterodimer to repress Myc-Max shared target genes) by differentiation inducers such as ATRA augments myeloid differentiation apparently by inhibiting E6AP.

### MNT overexpression arrests myeloid cells in G0-G1 phase and promotes myeloid differentiation

**Figure 4:**
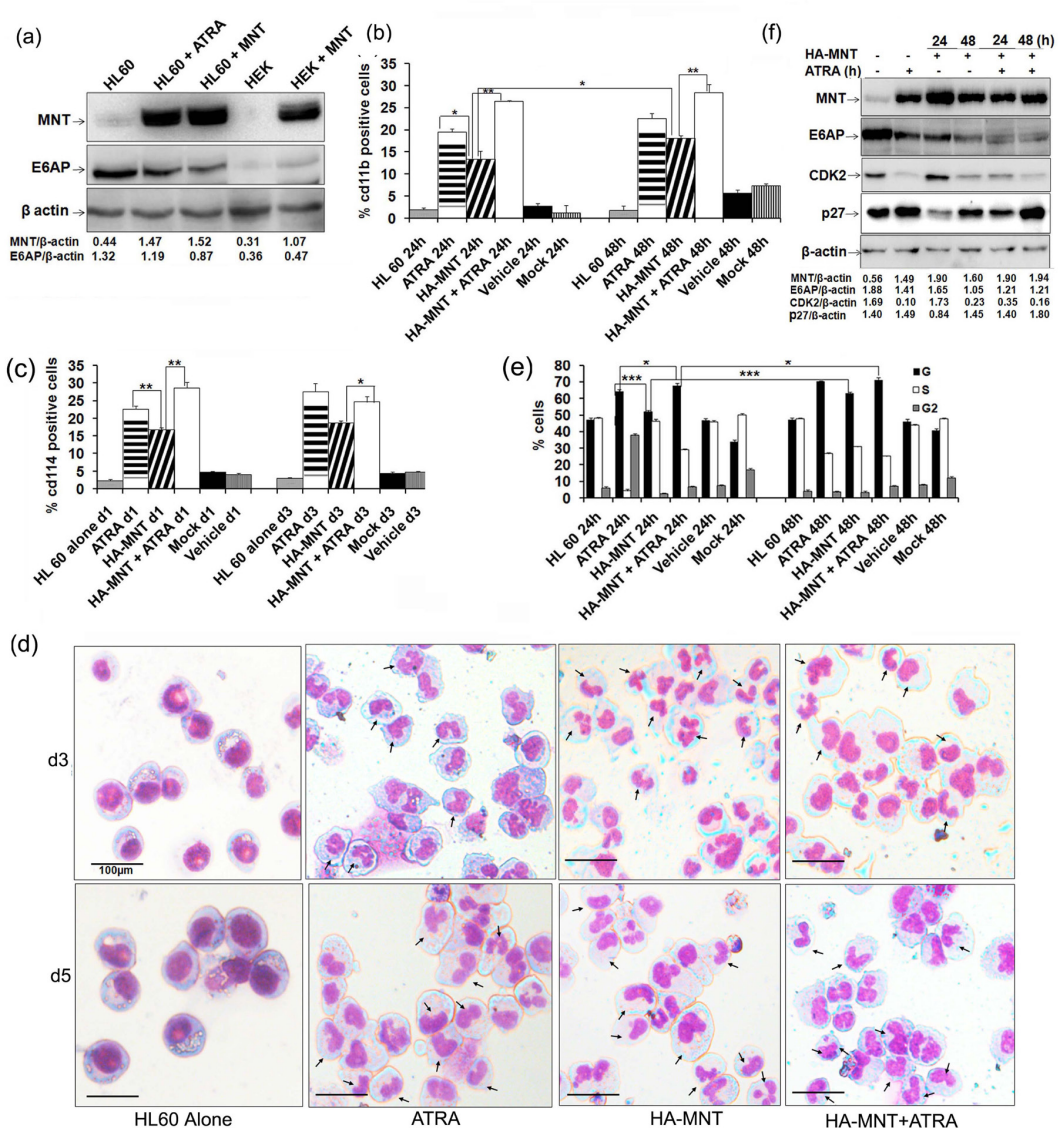
MNT overexpression promotes differentiation of HL60 cells **a.** HL60 cells were transiently transfected with 1.0μg HA-MNT and WCEs was prepared post-48h transfection. Lysates were resolved on 8% SDS-PAGE followed by immunoblotting with anti-MNT, E6AP and β-actin antibodies. **b.** Graphical representation of FACS analysis of cd11b in HL60 cells transiently transfected with HA-MNT (1.0μg) and co-treated with 1μM ATRA for 24 and 48h. **c.** Graphical representation of FACS analysis of cd114 in HL60 cells transiently transfected with HA-MNT (1.0μg) and co-treated with 1μM ATRA post 24h transfection. **d.** HA-MNT overexpression leads to granulocytic differentiation in HL60 cells. Cells were stained with May-Grunwald and Wright's Giemsa staining. Arrows indicate granulocytes/neutrophils. Scale represent 100μm. **e.** Graphical representation of percentage of G0/G1 cells post HA-MNT (1.0μg) transfection and co-treatment with ATRA post-36h transfection. **f.** HA-MNT over expressing HL60 cells were immunoblotted with antibodies against indicated proteins. Data are representative of minimum three independent experiments. Results are given as standard error of mean (+S.E.M.); *P<0.05, **P<0.001, ***P<0.0001. One-way ANOVA with Bonferroni's Multiple Comparison Test was performed using GraphPad Prism Version 5.00.

As we observed enhanced expression of MNT during myeloid differentiation, we next asked whether MNT overexpression can promote myeloid differentiation. To address this, HL60 cells transiently transfected with HA-MNT (Figure 4a) were assessed for cd11b (a general myeloid differentiation marker) (Figure 4b; representative 2D-dot plot for cd11b as assessed by FACS flow cytometry is shown in Supplementary Figure S2a) and cd114 (Figure 4c; respective 2D-dot plot for cd114 is shown in Supplementary Figure S2b) positivity post-indicated time points under FACS analyzer. Increased expression of both differentiation markers in HA-MNT transfected condition was observed. Besides assessing for surface proteins, we also performed May-Grunwald Wright's-Giemsa staining of cytospun cells to morphologically assess differentiation of these cells. Granule-like nuclear morphology in cells transfected with HA-MNT alone as well as co-treated with ATRA was observed (Figure 4d; day 3). Moreover, by day 5, cells attained myelocyte morphology with oval-shaped nucleus and obvious differentiation-like morphology (Figure 4d). Consistent with these findings in HL60 cells, overexpression of HA-MNT also triggered myeloid differentiation in U937 cells (Figure S3) both in a time-and dose-dependent manner. Together, these results indicated that MNT overexpression is sufficient to trigger myeloid differentiation, driving HL60 cells towards granulocyte lineage.

Furthermore, majority of the known myeloid differentiation inducers are known to arrest cells in G0-G1 phase [25-27] and because MNT also induces growth arrest [28]; we next investigated if MNT-induced differentiation is coupled with cell cycle arrest. To address this, we overexpressed HA-MNT (1.0μg) in serum starved HL60 cells for 0, 24 and 48h and performed PI staining to assess distribution of cells in different phases of the cell-cycle by FACS flow cytometry as previously described [12, 29]. HA-MNT overexpression in HL60 cells showed more number of cells to be arrested in G0-G1 phase post-48h transfection (64.50%). Strikingly, the percentage of cells increased to 72.16% in HA-MNT transfected cells co-treated with ATRA, suggesting MNT promoted growth arrest in HL60 cells (Figure 4e; representative cell cycle analysis as assessed by FACS is shown in figure S4.a). No significant change was observed in cells treated with DMSO as a vehicle. To explore HA-MNT induced cell cycle arrest in HL60 cells, specific cell-cycle regulatory proteins required for G0-G1/S transition were assessed by western blot analysis. In line with ATRA induced cell-cycle arrest of human myeloid cell lines [14, 30], overexpression of HA-MNT led to decrease in levels of CDK2 while increase in p27^Kip1^ levels post-48h transfection. Notably, the relative levels of CDK2 also decreased, while p27^Kip1^ levels increased in HA-MNT transfected condition co-treated with ATRA post-48h transfection (Figure 4f). The negligible repressive effects of MNT on these proteins at early time point (24h of MNT transfection alone) might be attributed to the lack of differentiation signal, which when provided through ATRA did repress these proteins even at 24h. Consistent with these findings in HL60 cells, overexpression of HA-MNT also induced G0-G1 arrest in U937 cells in time-dependent manner (as detailed in figure results S4.b and c). Together, these findings suggested that not only MNT knockdown inhibited myeloid differentiation; when overexpressed it may induce growth arrest and promote differentiation in these cells.

### MNT knockdown inhibits myeloid differentiation and promotes proliferation

**Figure 5:**
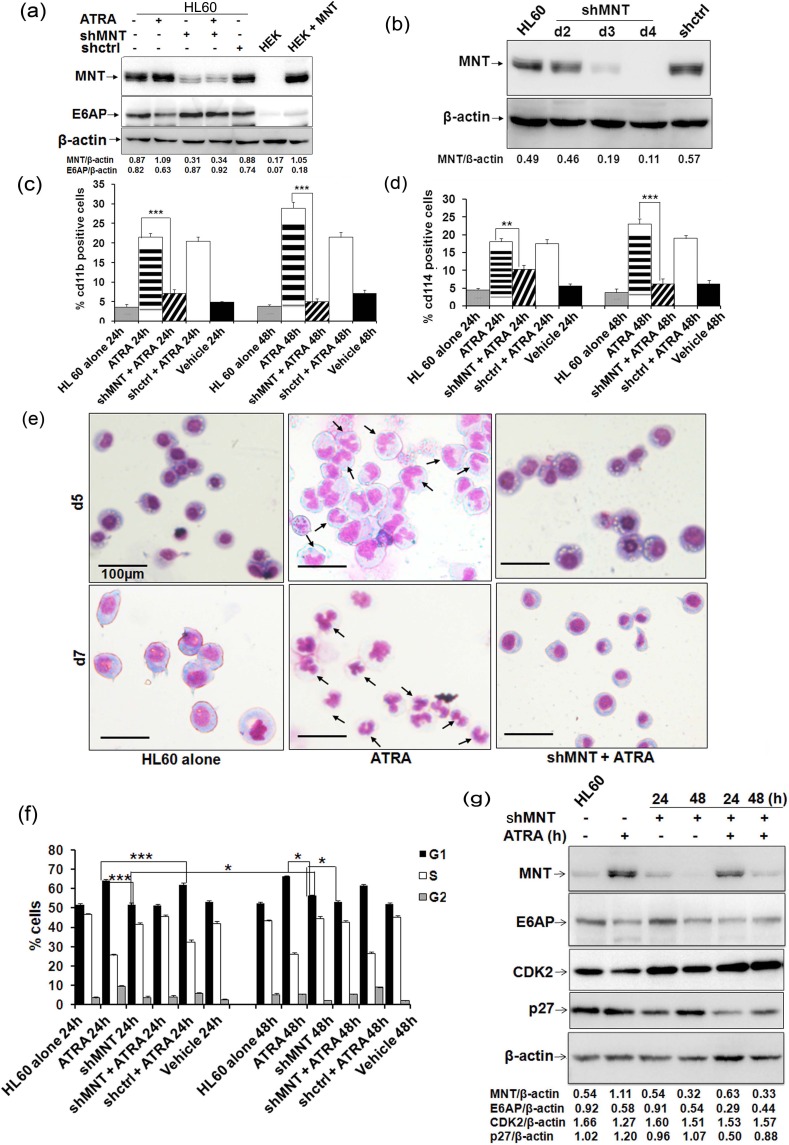
MNT knockdown inhibits myeloid differentiation and rescues G0-G1 arrest **a.** HL60 cells were transiently transfected with shMNT (1.0μg) and WCEs was prepared; cells were treated with 1μM ATRA post-36h transfection as indicated. Lysates were resolved on 8% SDS-PAGE followed by immunoblotting with MNT, E6AP and β-actin antibodies. **b.** HL60 cells were stably transduced with shMNT lentiviral vector and WCEs were prepared post-2, 3 and 4 days of transduction. Lysates were resolved on 8% SDS-PAGE followed by immunoblotting with anti-MNT antibody. **c.** Graphical representation of FACS analysis of cd11b in HL60 cells transiently transfected with shMNT (1.0μg) and co-treated with 1μM ATRA post-36h transfection. **d.** Graphical representation of FACS analysis of cd114 in HL60 cells transiently transfected with shMNT (1.0μg) and co-treated with 1μM ATRA post-36h transfection. **e.** MNT knockdown in HL60 cells *via* lentiviral transduction of shMNT inhibits granulocytic differentiation. Cells were stained with May-Grunwald and Wright's Giemsa staining. Arrows indicate granulocytes/neutrophils. Scale represent 100μm. **f.** Graphical representation of G0-/G1 cells post-24 and 48h shMNT (1.0μg) transfection and co-treatment with 1μM ATRA post-36h transfection. **g.** Effects of MNT knockdown on the protein levels of G1/S transition regulators in HL60 cells. WCEs were harvested post-24 and 48h shMNT (1.0μg) transfection and co-treated with 1μM ATRA post-36h transfection as indicated. Lysates were subjected to immunoblotting with antibodies against indicated G1/S transition regulatory proteins. Data are representative of minimum three independent experiments. Results are given as standard error of mean (+S.E.M.); *P<0.05, **P<0.001, ***P<0.0001. One-way ANOVA with Bonferroni's Multiple Comparison Test was performed using GraphPad Prism Version 5.00.

Because we observed profound MNT expression during ATRA mediated myeloid differentiation, we next sought to determine the effects of MNT knockdown in the process of differentiation. For this, we transiently transfected HL60 cells with control shRNA and shMNT (1.0μg); post-48h transfection, immunoblotting with MNT, E6AP and β-actin antibodies revealed persistent decrease in MNT protein levels (in shMNT transfected lanes; Figure 5a). Notably, even ATRA could not restore MNT protein levels in MNT knocked down condition. We also stably knocked down MNT through transduction of shMNT lentiviral particles and assessed the level of knock down and subsequent effects on myeloid differentiation. Immunoblotting with anti-MNT antibody showed efficient knock down of endogenous MNT in WCEs of HL60 cells post-3 and 4 days of transduction (Figure 5b). Further, this MNT knockdown substantially inhibited myeloid differentiation even in ATRA treated cells as shown by FACS analysis for cd11b (Figure 5c; Figure S5.a depicts the representative 2D-dot plot for cd11b) and cd114 expression (Figure 5d; Figure S5.b shows the representative 2D-dot plot for cd114) in HL60 cells transfected with shMNT (1.0μg) for 0, 24 and 48h; suggesting enhanced expression of MNT is required for induction of myeloid differentiation, while its knockdown is sufficient enough to inhibit even ATRA-mediated differentiation. Besides assessing expression of myeloid differentiation related surface proteins, we also monitored nuclear morphology in these MNT knocked down cells using May-Grunwald Wright's-Giemsa staining post-5 and 7-days of lentiviral transduction of shMNT (Figure 5e). Furthermore, MNT knockdown leading to inhibition of myeloid differentiation as measured by cd11b expression in yet other myeloid cell lines U937 and 32Dcl3 [23] (Figure S6) yet again strengthened our finding that MNT is required for induction of myeloid differentiation. Together, these results indicated that MNT knock down inhibited myeloid differentiation, a common phenomenon observed in several leukemia subtypes while its induced expression (such as *via* ATRA treatment) triggered myeloid differentiation.

Given the fact that MNT loss triggers proliferation [18], we next investigated distribution of cells in different phases of the cell cycle upon MNT knockdown. Serums starved HL60 cells transiently transfected with shMNT (1.0μg) and co-treated with ATRA were assessed for cell cycle distribution after 0, 24 and 48h. Post-indicated time points, cells stained with PI and analyzed in flow cytometer for DNA content profile revealed substantial decrease in the number of cells (post-48h MNT knockdown *via* shMNT) in G0-G1 phase (55.14%) compared to cells treated with ATRA for 48h (67.09%). Interestingly, co-treatment with ATRA for further 48h post-36h shMNT transfection (Figure 5f; representative cell cycle analysis is shown in Figure S5.c) could not rescue G0/G1 growth arrest (51.88%) in comparison to cells treated with ATRA only (67.09%), suggesting MNT knockdown augments proliferation and rescues ATRA-mediated cell-cycle arrest in HL60 cells.

To substantiate MNT knockdown mediated rescue of growth arrest, we performed western blotting to evaluate the changes in the expression of specific cell-cycle regulatory proteins for 24 and 48h (Figure 5g). MNT knockdown led to increase in CDK2 while decrease in p27 ^Kip1^ levels post-24 shMNT transfection compared to cells treated with ATRA only (Figure 5g). Notably, even ATRA treatment was unable to revert expression of these regulatory proteins in MNT knockdown cells (particularly 48h knockdown condition); suggesting MNT to be a molecular mediator of ATRA induced growth arrest in myeloid cells.

### Inhibition of E6AP promotes myeloid differentiation with G0-G1 growth arrest in HL60 cells

**Figure 6:**
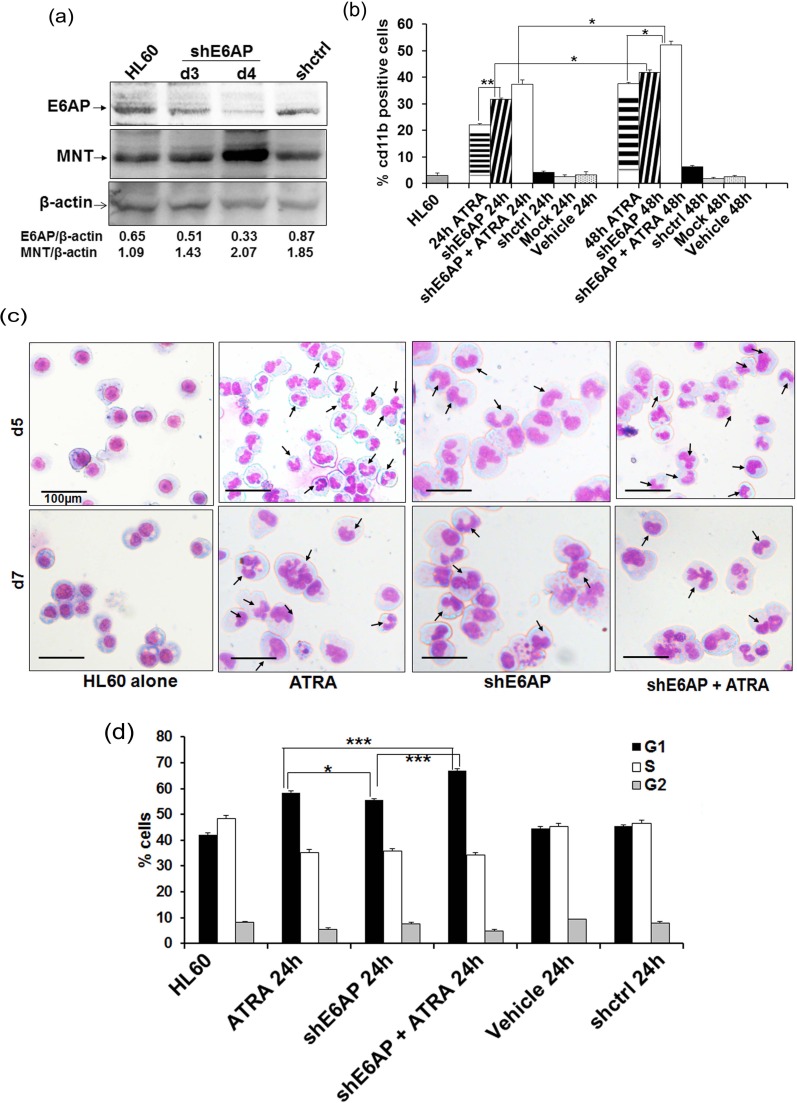
shE6AP mediated E6AP inhibition enhances MNT expression and promotes differentiation in HL60 cells **a.** HL60 cells were transduced with lentiviral shE6AP vector and WCE was prepared post-3 and 4 days of transduction. WCEs were resolved on 10% SDS-PAGE and immunoblotted with anti-MNT, E6AP and β-actin antibodies. **b.** HL60 cells were transfected with shE6AP as indicated. Cells were washed and labelled with cd11b and its respective IgG-PE conjugated antibody for FACS analysis. **c.** E6AP knockdown *via* lentiviral transduction of shE6AP leads to granulocytic differentiation in HL60 cells. After indicated time points, cells were stained with May-Grünwald and Wright's Giemsa Arrows indicate granulocytes/neutrophils. Scale represent 100μm. **d.** Graphical representation of percentage of G0/G1 cells after knockdown of endogenous E6AP and co-treated with 1μM ATRA post-24h transfection. Data are representative of minimum three independent experiments. Results are given as standard error of mean (+S.E.M.); *P<0.05, **P<0.001, ***P<0.0001. One-way ANOVA with Bonferroni's Multiple Comparison Test was performed using GraphPad Prism Version 5.00.

As we observed inverse levels of MNT and E6AP expression during myeloid cell differentiation and showed that E6AP targeted MNT for ubiquitin-mediated proteasome degradation, we next sought to determine the functional consequences of E6AP knockdown in myeloid cells [13]. E6AP knockdown in stably transduced HL60 cells with shE6AP lentiviral particles restored MNT protein levels post-4-days infection (Figure 6a) and triggered myeloid differentiation as shown by enhanced cd11b positivity; 42.88% cells showed cd11b expression compared to 5.85% in shCtrl infected cells (Figure 6b; representative FACS 2D-dot plot is shown in figure S7.a). This clearly demonstrated that E6AP knockdown leading to MNT restoration promoted myeloid differentiation. Also as expected, E6AP knockdown furthered and augmented ATRA induced myeloid differentiation (cd11b expression increased from 38.02% to 53.68%). Consistent with FACS analysis, post-5 and 7 days of culture, morphological examination of these stably transduced HL60 cells by May-Grunwald Wright's-Giemsa staining also showed cells with multi-lobed nuclei and some with eccentric and indented nuclei, a feature of differentiation (Figure 6c). Also, given the fact that differentiation parallels G0-G1 growth arrest of cells, we investigated the kinetics of E6AP inhibition-induced cell-cycle arrest by flow cytometric analysis of PI-labelled nuclei. HL60 cells were transfected with shE6AP (1.0μg) and co-treated with ATRA for 0 and 24h and cell-cycle distribution was analyzed (Figure 6d) as previously described [29]. Cells started to accumulate in the G0/G1 phase of the cell-cycle after 24h of transfection (56.38%) and the percentage of G0/G1 cells substantially increased in cells transfected with shE6AP and co-treated with ATRA (68.31%) in contrast to shcontrol (59.01%) (Figure 6d; representative cell cycle analysis is shown in figure S7.b), suggesting E6AP knockdown augments ATRA mediated growth arrest leading to increased myeloid differentiation. Taken together, these data demonstrate that E6AP inhibition stabilizes MNT protein levels, which in turn may lead to enhanced myeloid differentiation coupled with G0-G1 growth arrest.

## DISCUSSION

In the present study, using GST pull down approach coupled with proteomics based mass spectrometry we have identified MNT as a novel interacting partner of E6AP. Here we showed E6AP physically interacted with MNT in myeloid cells (Figure 1) and promoted its ubiquitin mediated proteasome degradation, whereas catalytically inactive mutant E6APC843A rather stabilized MNT (Figure 2). Our data demonstrated that overexpression of E6AP in myeloid cells decreased the steady state expression of MNT while treatment with proteasomal inhibitors MG132 and Lactacystin restored its expression. On the contrary, catalytically inactive mutant E6APC843A failed to promote degradation of MNT confirming ligase activity of E6AP to be required for down regulation of MNT *via* ubiquitin-mediated proteasome degradation (Figure 2).

Reportedly, MNT loss triggered Myc transcription targets, proliferation, apoptosis and transformation [18, 31]. In line with these reports that suggested MNT to play a role in cellular differentiation and cell cycle control; we also investigated MNT expression and function during terminal differentiation of myeloid cells with respect to E6AP. We took advantage of two well characterised cell lines that undergo defined programs of myeloid differentiation. HL60 is a pluripotential promyelocytic cell line that differentiates into granulocytes when treated with ATRA [32] and into macrophages by PMA [33]. U937 is a promonocytic cell line that can be induced to differentiate into macrophages by PMA [34] or vitamin D_3_ [22]. We observed enhanced expression of MNT in nuclear fractions with concomitant decrease in E6AP while an inverse expression in the cytoplasmic fractions of these cells (HL60/U937) induced to differentiate either by PMA, ATRA or Vitamin D_3_ (Figure 3). Similar patterns of MNT and E6AP expression also observed during cytokine (G-CSF) induced differentiation of 32Dcl3 cells further indicated that MNT upregulation is a general phenomenon during granulopoiesis (Figure 3). Notably, 32Dcl3 cells undergo terminal differentiation by G-CSF. IL-3 replacement with G-CSF stimulates proliferation of these cells for 4-5 days followed by growth arrest and appears neutrophil-like morphology by day 12 [13, 23]. Because like MG132, ATRA also restored MNT expression even in E6AP overexpressed HL60 cells (Figure 3b), it is likely that these differentiation inducers inhibited E6AP-mediated ubiquitination of MNT and thereby stabilized it (Figure S1b). Although further studies are required to unveil the mechanisms of E6AP inhibition by these inducers, another possible mechanism may be induction of E6AP autodegradation by these inducers as we observed ATRA substantially inhibited even overexpressed E6AP in HL60 cells (Figure 3b). Moreover, previous reports also showed that autoubiquitination and subsequent degradation of E6AP represented a mechanism to control intra-cellular E6AP levels [35]. In addition, differentiation inducer-mediated induction and activation of specific Deubiquitinases (DUBs) that inhibited E6AP-mediated ubiquitination of substrates might be another possibility [36]. Our study also uncovered a novel role for MNT to promote myeloid differentiation which otherwise was targeted for degradation by E6AP. Our hypothesis was strengthened by set of observations demonstrating upregulation of MNT expression with concomitant down regulation of E6AP in myeloid cells treated with ATRA and other differentiation inducing agents in a time-dependent manner (Figure 1c and Figure 3a, 3c, 3d, 3e and 3f). Moreover, MNT overexpression promoted G0-G1 growth arrest and drove cells to undergo myeloid differentiation (Figure 4, Supplementary Figure S2, S3 and S4), while its knockdown was sufficient enough to mitigate either ATRA or PMA induced myeloid differentiation in HL60 (Figure 5 and Supplementary Figure S5) and U937 cells (Supplementary Figure S6b and c), respectively. Degradation of MNT apparently by E6AP leading to inhibition of myeloid differentiation was substantiated by the observation that knockdown of E6AP enhanced MNT expression in myeloid cells and promoted its differentiation (Figure 6 and Supplementary Figure S.7). Together, these findings supported the notion that MNT is a key mediator of myeloid differentiation. In fact, a previous report showing MNT overexpression in neuroblastoma cells retained differentiation potential [37] also augments and supports our findings that MNT has a pivotal role in differentiation per se.

Our data also warrant for enhanced MNT expression to be a pre-requisite for induction of myeloid differentiation. This is mainly because enhanced MNT possibly may be required for formation of heterodimeric Mnt-Max complex in the nucleus to arrest cells in G0-G1 phase and thus help cells to exit the cell cycle to differentiate by repressing expression of proliferation promoting genes [24]. In line with this, induction of growth arrest and myeloid differentiation by restored MNT due to E6AP inhibition by ATRA may thus be attributed to the increased Mnt-Max repressive complex formation and thus limiting Max availability for Myc to form the active Myc-Max complex. Intact interaction between Mnt-Max during myeloid differentiation as observed by indirect immunofluorescence and Co-immunoprecipitation (Supplementary Figure S1c and S1d) consolidated our assumption that ATRA or other differentiation inducers by enhancing formation of Mnt-Max complex repressed expression of (Myc-Max) shared targets genes [16] involved in proliferation. Thus, causing growth arrest and providing a window for cells to exit the cell-cycle and undergo differentiation. As loss of MNT is associated with tumorigenesis and transformation [17], our results predict a functional correlation between elevated levels of E6AP and reduced MNT expression in myeloblasts. Although detailed investigation is required to determine the formation of Myc-Max/Mnt-Max complexes during myeloid differentiation. Nonetheless, our finding showing inhibition of E3 ubiquitin ligase (E6AP) leading to enhanced half-life of MNT was consistent with previous finding showing reduced half-life of MNT to be responsible for increased Myc-Max complex formation and expression of target (proliferative) genes [24]. Taken together, our data demonstrate that restored MNT may limit Max availability for Myc-Max complex formation leading to growth arrest. Although not much is known about transcription factors that may regulate MNT during granulopoiesis, it is possible that transcription factors such as C/EBPα that couple growth arrest with differentiation in myeloid cells may regulate MNT expression. In fact, in our previous study we have also identified MNT as a putative interacting partner of C/EBPα during granulocytic differentiation in myeloid cells [38]. Moreover, C/EBPα is a key regulator of granulopoiesis and executed its function by coupling the direct transcriptional activation of tissue-specific genes with the arrest of cell proliferation [39, 40]. Furthermore, since ectopic/conditional expression of C/EBPα is known to trigger neutrophilic differentiation of myeloid cells [41], we observed that overexpression of C/EBPα in K562 myeloid cells led to transient increase in the endogenous levels of MNT post-48h transfection (Figure S8), thus providing an indirect evidence for MNT regulation by C/EBPα. However, it requires further studies to investigate the precise regulation of MNT by myeloid specific transcription factors during granulopoiesis.

Taken together, here we identified MNT as a novel interacting partner of E6AP and demonstrate a unique regulatory role for MNT in governing key functions in myeloid cells associated with growth arrest and cellular differentiation. Our data showed that E6AP negatively regulated granulocytic differentiation by targeting MNT for degradation. Further, myeloid cell differentiation inducers including ATRA seem to exploit MNT for inducing growth arrest and subsequent myeloid differentiation by inhibiting E6AP and promoting formation of Mnt-Max complex over Myc-Max. Thus, targeting E6AP or enhancing expression of MNT can have therapeutic implications in rescuing myeloid differentiation in leukemia and other cancers where MNT is a potential transcriptional repressor.

## MATERIALS AND METHODS

### Cell culture, plasmids and transient transfection

HL60, U937, K562, 32Dcl3 and HEK293T cells obtained from ATCC were cultured as previously described [13, 23, 29, 42]. siMNT (Smartpool: ON-TARGET plus MNT SiRNA L-009373-00-0010), scrambled siRNA and siRNA transfection reagent DhermaFECT (ThermoScientific T-2001-03) were purchased from Dhermacon RNA technologies (Lafayette, CO, USA). shMNT (TRCN00000234786), shE6AP (TRCN0000235499) and shRNA control were purchased from Sigma. Expression plasmids for HA-MNT [43], pcDNA3.1-E6AP [10], pCAG-HA-E6AP-C843A [44], pGEX4T-GST-E6AP [45] were kind gifts from G. Meroni, Nihar Jana, Ikuo Shoji and Zafar Nawaz, respectively. Transfections in adherent cells were performed using Lipofectamine-LTX and Plus Reagent (15338-100; Invitrogen) as per manufacture`s protocol as previously described [13, 42]; Transfections in myeloid cells HL60 and U937 were performed using AMAXA cell Nucleofector kit V (VCA-1003; Lonza). E6AP-C843A-HA is a catalytically inactive mutant of E6AP [13]. Cells were treated with 10μM MG132 (Z-Leu-Leu-Leu-al, Sigma, St.Louis, MO) and 10μM Lactacystin (Sigma) 6h prior to cell lysis.

### GST pull-down and SDS-PAGE

GST and GST-E6AP were expressed in *E.Coli* with 0.2mM IPTG induction at 20°C for 16h and purified as described before [42, 46]. HL60 WCEs treated with ATRA were prepared using RIPA buffer. Equal amounts of GST and GST-E6AP were incubated with 1mg of WCEs in NETN buffer as described previously [46].

### MALDI-TOF/TOF analysis

MALDI-TOF/TOF analysis was performed as described previously [12, 29, 46]. Briefly, GST-E6AP interacting proteins were excised, destained with 50mM ammonium bicarbonate-acetonitrile (1:1) for 15 min followed by 2 alternating washing steps with 50% acetonitrile and 50mM ammonium bicarbonate. The gel pieces were dehydrated at room-temperature with 100% acetonitrile and covered with 10μl of trypsin (working solution 5ng/μl prepared from 100ng/μl stock solution in 50mM ammonium bicarbonate) overnight at 37°C. The peptides were eluted in 10μl of 70% acetonitrile. The eluates were dried using a vacuum centrifuge and stored at −80°C. The peptides were resuspended in 5 μl of 20% acetonitrile and 0.1% TFA and sonicated for 3 min before processing for mass spectrometry. MALDI-TOF/TOF data was analyzed with ProteinPilot^TM^ Software (AB Sciex) using MASCOT search engine. The peptide mass data were analyzed for corresponding protein matching in the Swiss-Prot database with settings of peptide mass tolerance: +150ppm, fragment mass tolerance: +0.3Da and MAX missed cleavages set as 1 in MS/MS ion search using MASCOT search engine. Proteins that could be identified with more than 2 peptides had a % confidence index >99%, while the one with one peptide had a % confidence index >95% [42, 46].

### Immunoblotting

Post-transfection, cells were lysed with RIPA buffer as previously described [13]. Antibodies used for anti-MNT (sc-376708), anti-GST (sc-459), anti-CDK2 (M-2) (sc-163), PCNA (C-20) (sc-9857), Lamin A (H-102) (sc-20680) and β-actin (sc-47778) were purchased from Santa Cruz Biotechnology Inc; anti-HA (2367S), anti-p27 ^Kip1^ (2552S) and anti-His tag (2365S) from Cell Signalling Technology and anti-E6AP (E8655) from Sigma Aldrich. Chemiluminiscence was detected with ECL western blotting detection reagents (Millipore) using LAS 4010 (GE Healthcare) as previously described [13].

### Immunofluorescence microscopy

293T cells were plated in chamber slide one day before transfection. Next day, cells were transfected with E6AP and HA-MNT. Post-24h transfection cells were processed for immunofluorescence microscopy as described previously [23]. Alexa Fluor 594 donkey anti-rabbit (A21207; Invitrogen) and Alexa Fluor 488 goat anti-mouse (A11055; Invitrogen) secondary antibodies and 4′,6-diamino-2-phenylindole staining were procured from Invitrogen and Sigma-Aldrich, respectively. Cells were mounted with vectashield (Vector Laboratories, Burlingame, CA, USA) and visualized under fluorescence microscope (Leica, Wetzlar, Germany). HL60 cells treated with ATRA for 0 and 48h were cytospun and proceeded for immunostaining as above.

### Co-immunoprecipitation assay

Co-IP was performed as described previously [13, 23, 42, 46]. Briefly, For Co-IP cell lysates were prepared in RIPA buffer (1% (v/v) NP40, 0.5% (w/v) sodium deoxycholate, 0.1% (w/v) SDS, 0.15M NaCl, 5mM EDTA, 1mM DTT and protease inhibitors). Protein lysates after preclearing with IgG were incubated with anti-MNT or E6AP antibodies and Protein Agarose G or A beads for 3h at 4°C in IP buffer (1% TBS, 0.5% NP40, protease inhibitors. After incubation beads were washed with IP buffer three times and bound proteins were eluted in Laemmli buffer. Samples were separated on 10% SDS-PAGE and were subsequently immunoblotted with MNT and E6AP antibody [13].

### Flow cytometry analysis

Cell-cycle profile was analyzed using flow cytometry. Briefly, 2 × 10^6^ cells were washed with PBS, fixed in 80% ethanol and resuspended in 50μg/ml propidium iodide (Sigma) and 125U/ml RNase A (Sigma). For cell cycle profile, DNA content was analyzed by flow cytometry using FACScan and Cell Quest Software (Becton Dickinson, USA). For detection of cell differentiation antigen cd11b and cd114, 1 × 10^6^ cells were washed twice with PBS, incubated with PE-conjugated cd11b or cd114 antibody or PE-conjugated IgG isotype control antibody at 4°C for 30 min and analysed by flow cytometry using FACScan and Cell Quest Software (Becton Dickinson, USA) [12, 13, 29].

### May-Grunwald and Wright`s Giemsa staining

HL60 cells were cytospin on slides; air-dried and stained with May-Grunwald and Wright`s Giemsa solution as previously described [13, 23]. Briefly, cells were stained with May-Grunwald for 5 min followed by washing with 1X PBS for 2 min. Meanwhile, Wright`s Giemsa solution was diluted (1:20) in 1X PBS and slides were stained in this diluted solution for 15-20 min. Cells were then washed in running tap water to remove the excess stain, air-dried and subjected to microscopic examination under light microscope (20X). Scale bars represent 100μm [13].

### Lentiviral infection

293T cells were co-transfected using calcium-phosphate transfection with either shctrl or ShMNT (TRCN00000234786, Sigma)/ShE6AP (TRCN0000235499, Sigma), along with the packaging plasmids psPAX2 (Plasmid #12260, Addgene) and pMD2.G (Plasmid #12259, Addgene). Virus-containing supernatants were collected at 24, 48, 72 and 96h after transfection, filtered through a 0.45μm-filter and centrifuged at 50,000 rpm for 2h at 4°C in a Optima XPN-100 ultracentrifuge using a 70Ti rotor. Pellets were resuspended in sterile phosphate-buffered saline and stored at −80°C. Lentiviral supernatants were used to transduce cells in the presence of 8μg/ml Polybrene (Sigma). Transduction of HL60 cells was performed in 6-well culture dish and harvested post-indicated time points [47-49].

## SUPPLEMENTARY MATERIAL FIGURES AND TABLE


