# Supplementary material for: Proteomic discovery of MNT as a novel interacting partner of E3 ubiquitin ligase E6AP and a key mediator of myeloid differentiation
Source: Oncotarget. 2015 Oct 25;7(7):7640–56. doi: 10.18632/oncotarget.6156 (PMC4884944; doi:10.18632/oncotarget.6156)
Supplement: Supplementary file 1 [file oncotarget-07-7640-s001.pdf]

# Proteomic discovery of MNT as a novel interacting partner of E3 ubiquitin ligase E6AP and a key mediator of myeloid differentiation

## Supplementary Material

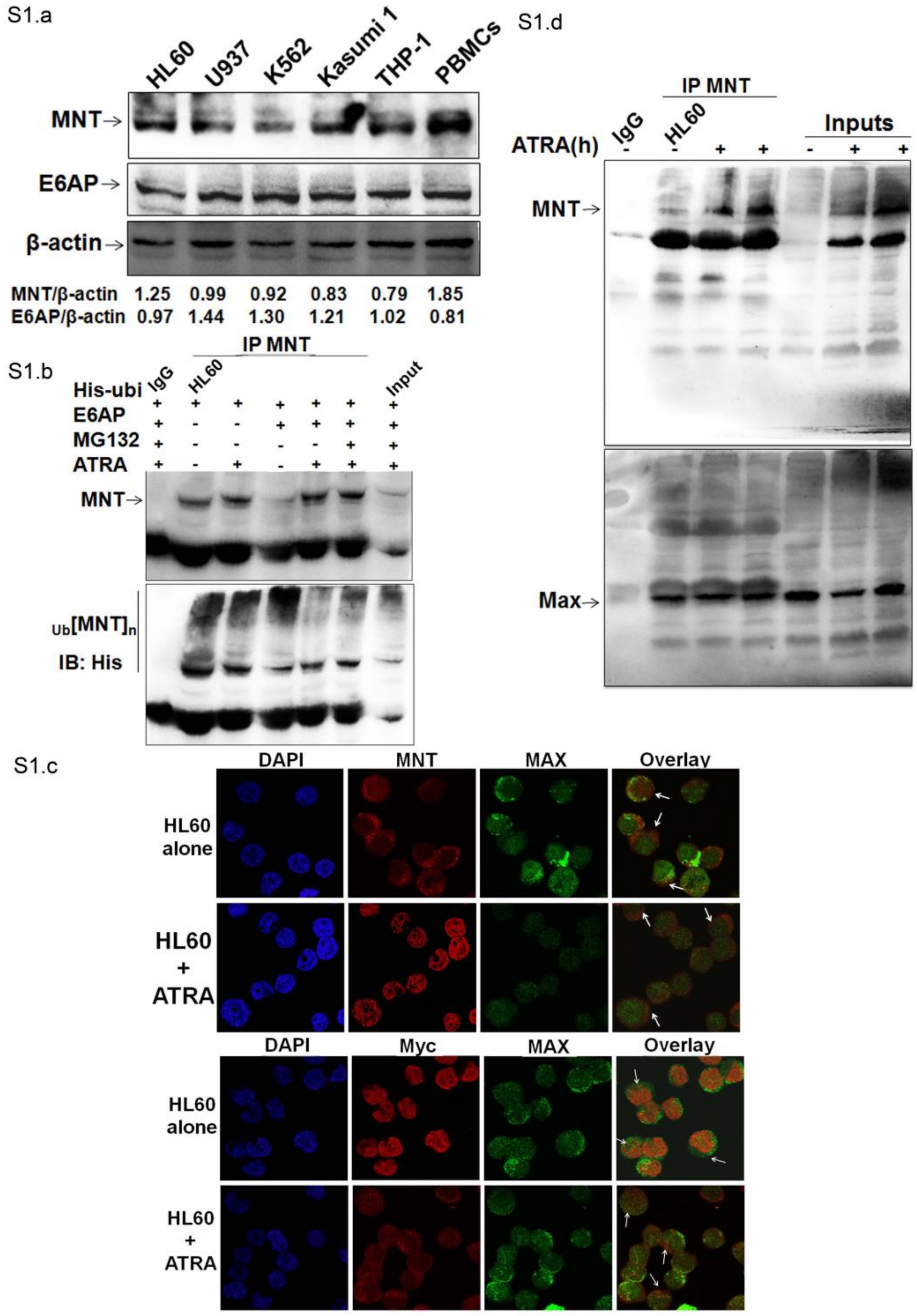

**S1: (a)** Immunoblotting for endogenous levels of MNT in myeloid leukemia cell lines. **(b)** HL60 cells were transfected with His-ubi (1.0µg) or E6AP (1.0µg) as indicated. Cells were also treated with ATRA and MG132 as indicated. MNT was co-immunoprecipitated and probed with anti-MNT antibody (upper panel). The same membrane was stripped and probed with anti-His antibody (lower panel). **(c)** indirect immunofluorescence staining for MNT, Max and Myc using respective conjugated secondary antibodies. The morphology of the cells was visualized under fluorescence microscope (63X). **(d)** Co-immunoprecipitation in HL60 cells treated with ATRA using anti-MNT antibody was performed and immunoblotting with anti-MNT followed by anti-Max antibody after stripping the same blot.

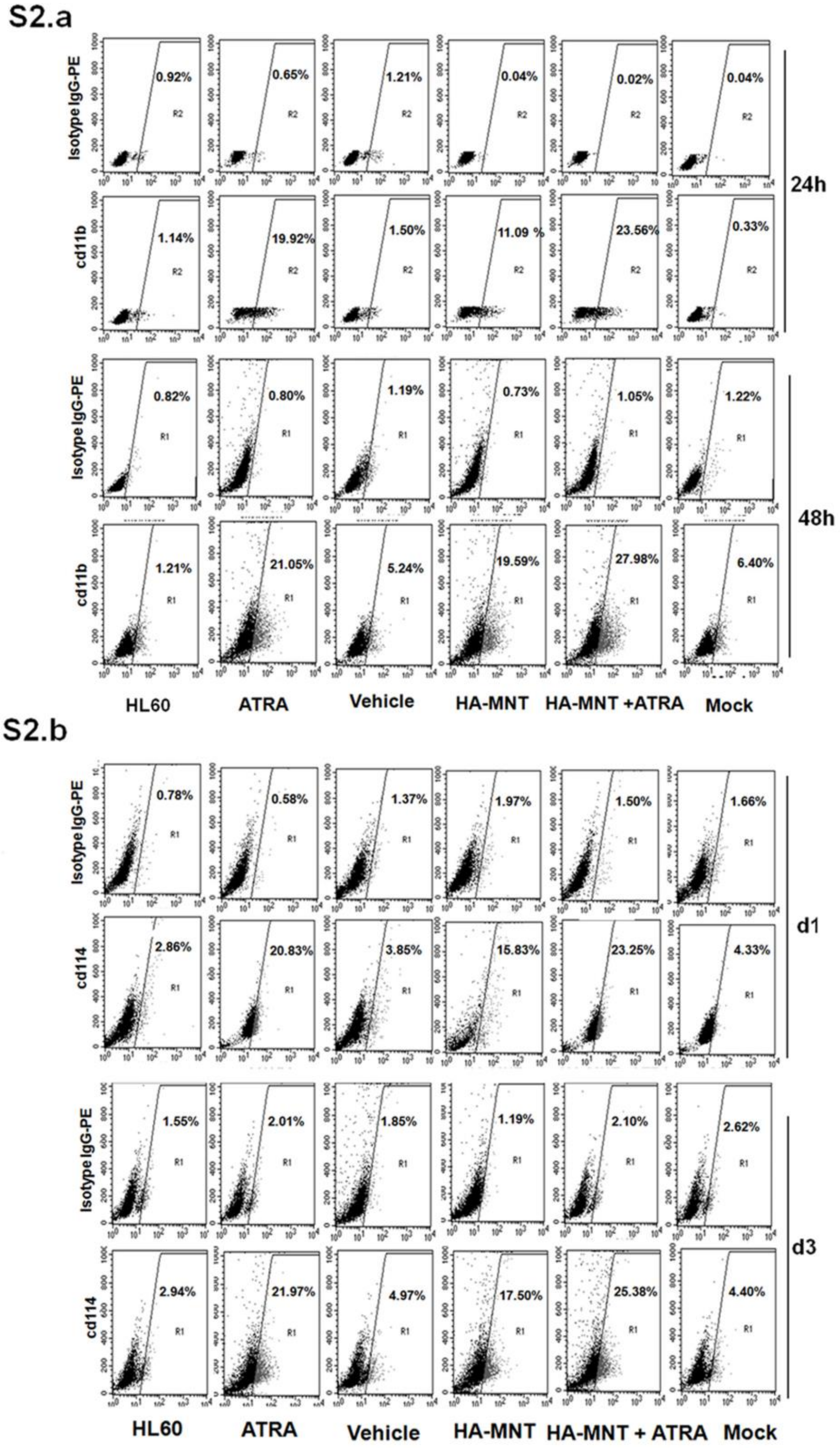


**S2: (a)** 2D-dot plot for FACS flow cytometry analysis of cd11b in HL60 cells transfected with HA-MNT post-24 and 48h. **(b)** 2D-dot plot for FACS flow cytometry analysis of cd114 in HL60 cells transfected with HA-MNT post-24 and 48h.

S3.a

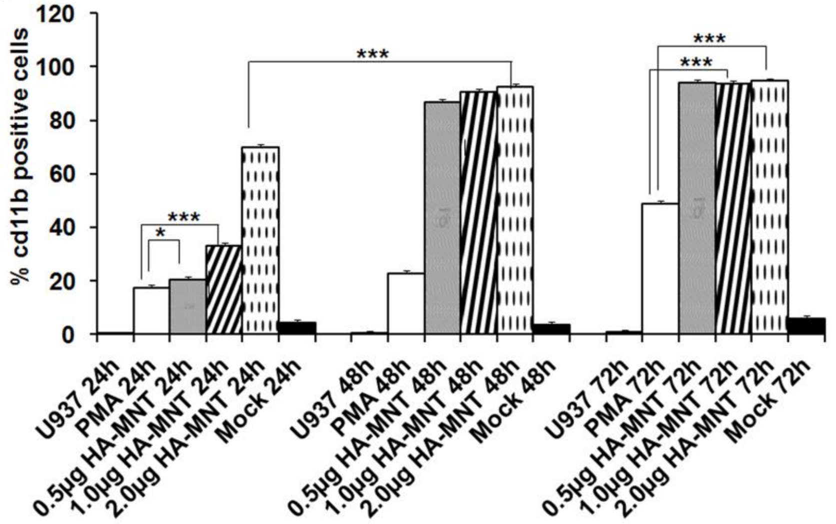


S3.b

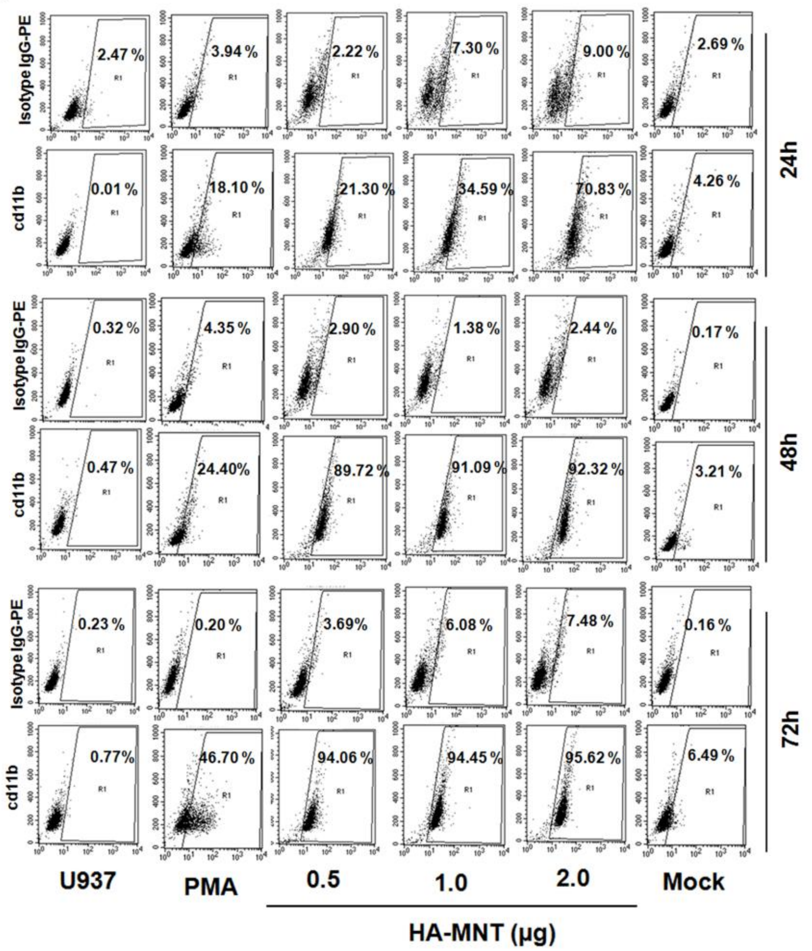


**S3: (a)** graphical representation of cd11b expression as assessed by FACS flow cytometry in U937 cells transfected with HA-MNT (0.5, 1.0 and 2.0µg) post-24, 48 and 72h transfection. **(b)** Representative 2D-dot plot for FACS analysis for cd11b expression in U937 cells transfected with HA-MNT (0.5, 1.0 and 2.0µg) post-24, 48 and 72h transfection.

S4.a

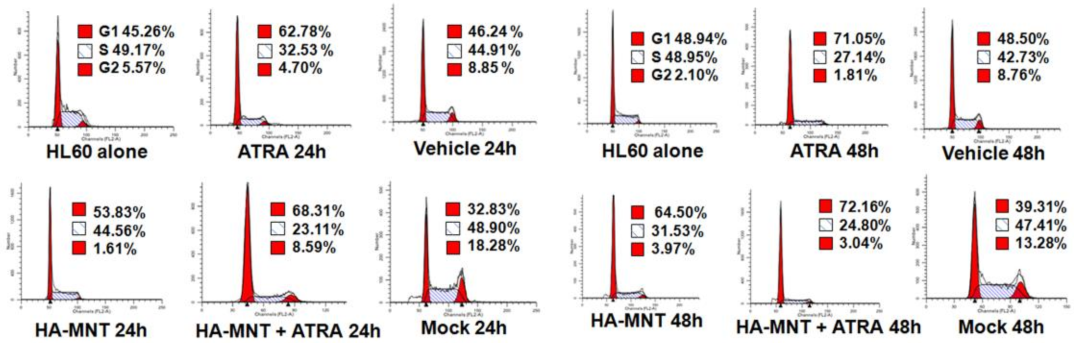


S4.b

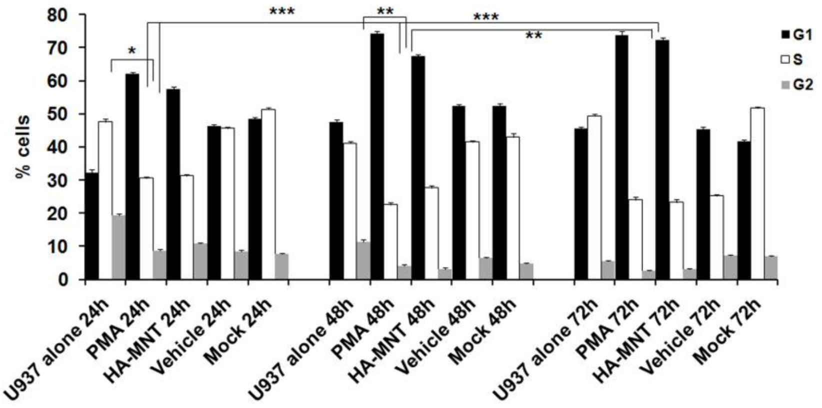


S4.c

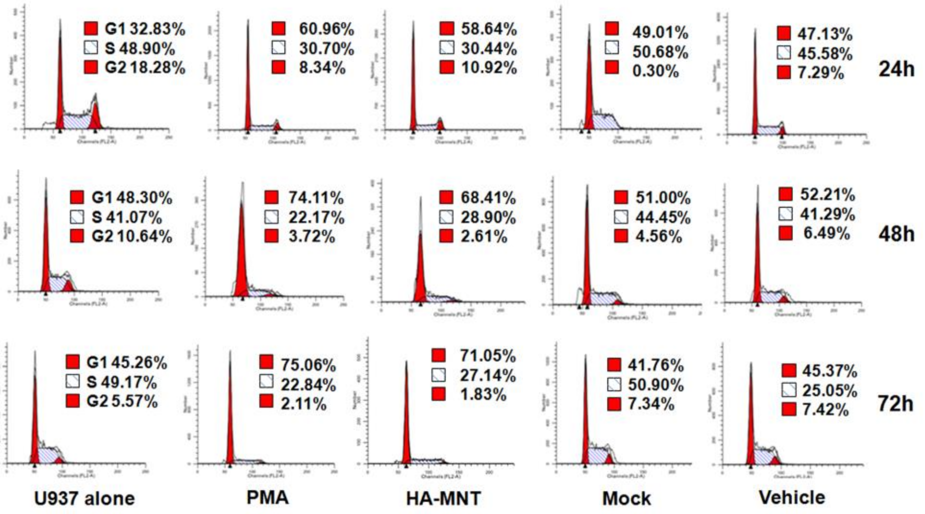


**S4: (a)** Representative cell cycle analysis of HL60 cells transfected with HA-MNT post-24 and 48h. **(b)** graphical representation of percentage of cells in different phase of the cell cycle as assessed by FACS flow cytometry in U937 cells transfected with HA-MNT (0.5) post-24, 48 and 72h transfection. **(c)** Representative FACS analysis of percentage of cells in different phase of the cell cycle in U937 cells transfected with HA-MNT (0.5µg) post-24, 48 and 72h transfection.

S5.a

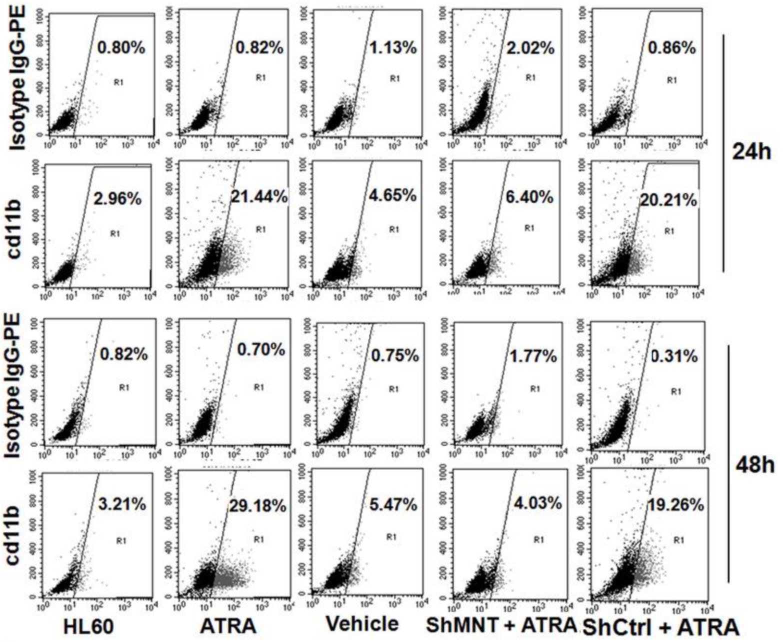
Isotype IgG-PE
cd11b
Isotype IgG-PE
cd11b
24h
48h
0.80%
0.82%
1.13%
2.02%
0.86%
2.96%
21.44%
4.65%
6.40%
20.21%
0.82%
0.70%
0.75%
1.77%
0.31%
3.21%
29.18%
5.47%
4.03%
19.26%
R1
HL60
ATRA
Vehicle
ShMNT + ATRA
ShCtrl + ATRA

S5.b

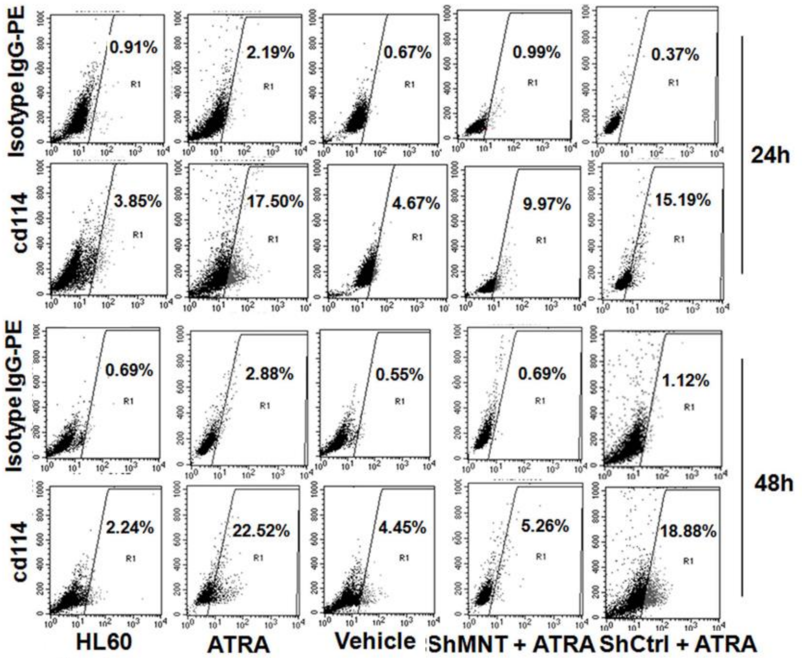
Isotype IgG-PE
cd114
Isotype IgG-PE
cd114
24h
48h
0.91%
2.19%
0.67%
0.99%
0.37%
3.85%
17.50%
4.67%
9.97%
15.19%
0.69%
2.88%
0.55%
0.69%
1.12%
2.24%
22.52%
4.45%
5.26%
18.88%
R1
HL60
ATRA
Vehicle
ShMNT + ATRA
ShCtrl + ATRA

S5.c

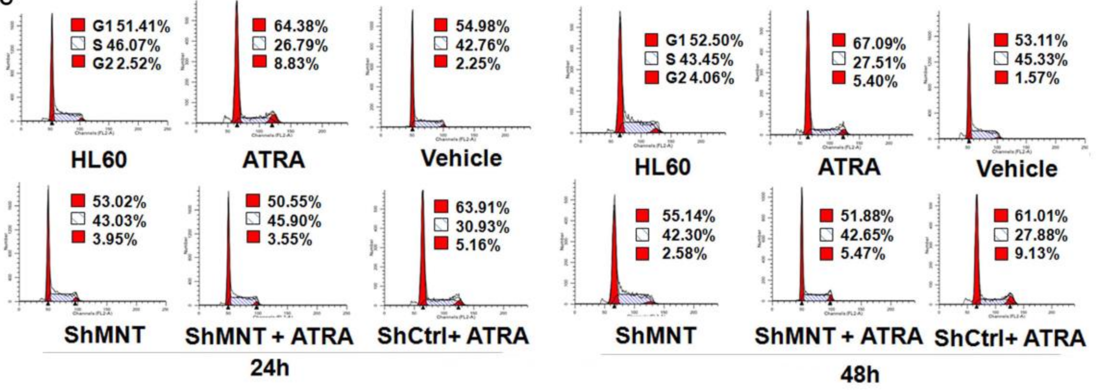
G1 51.41%
S 46.07%
G2 2.52%
64.38%
26.79%
8.83%
54.98%
42.76%
2.25%
G1 52.50%
S 43.45%
G2 4.06%
67.09%
27.51%
5.40%
53.11%
45.33%
1.57%
HL60
ATRA
Vehicle
HL60
ATRA
Vehicle
53.02%
43.03%
3.95%
50.55%
45.90%
3.55%
63.91%
30.93%
5.16%
55.14%
42.30%
2.58%
51.88%
42.65%
5.47%
61.01%
27.88%
9.13%
ShMNT
ShMNT + ATRA
ShCtrl+ ATRA
ShMNT
ShMNT + ATRA
ShCtrl+ ATRA
24h
48h

**S5: (a)** Representative 2D-dot plot for FACS analysis of cd11b expression in HL60 cells transfected with shMNT (1.0µg) and co-treated with ATRA for 0, 24, 48 and 72h. **(b)** Representative 2D-dot plot for FACS analysis of cd114 expression in HL60 cells transfected with shMNT (1.0µg) and co-treated with ATRA for 0, 24, 48 and 72h. **(c)** Representative cell cycle analysis for percentage of cells in different phases of the cell cycle in HL60 cells transfected with shMNT (1.0 µg) and co-treated with ATRA post-36h ShMNT transfection for further 0, 24 and 48h.

S6.a

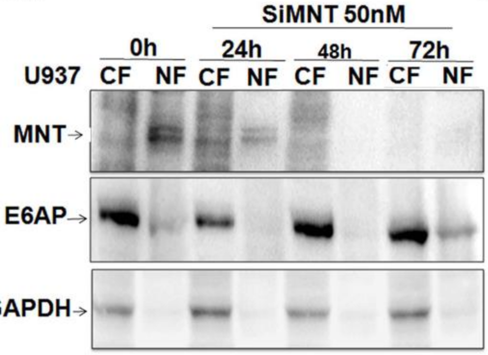


S6.b

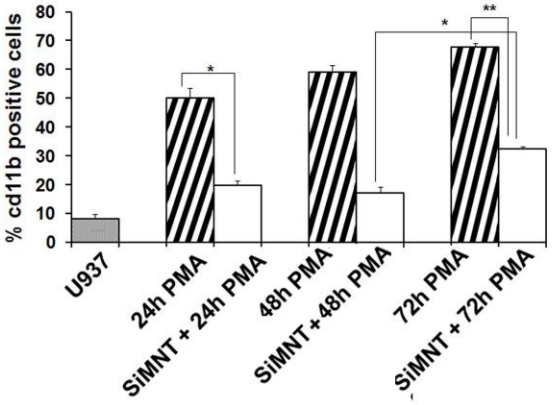


S6.c

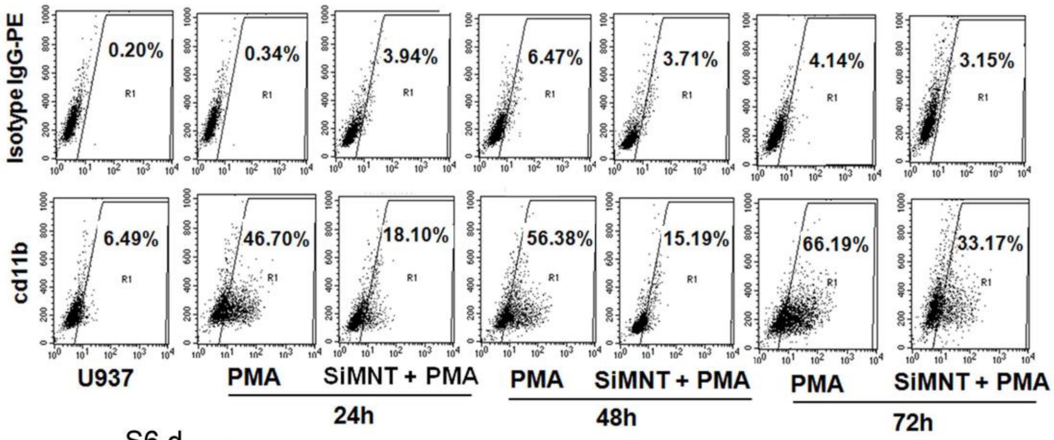


S6.d

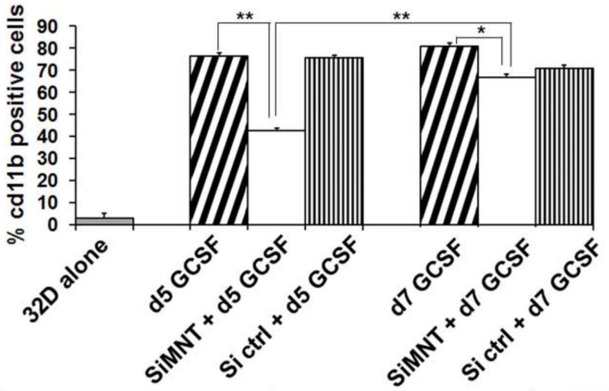


S6.e

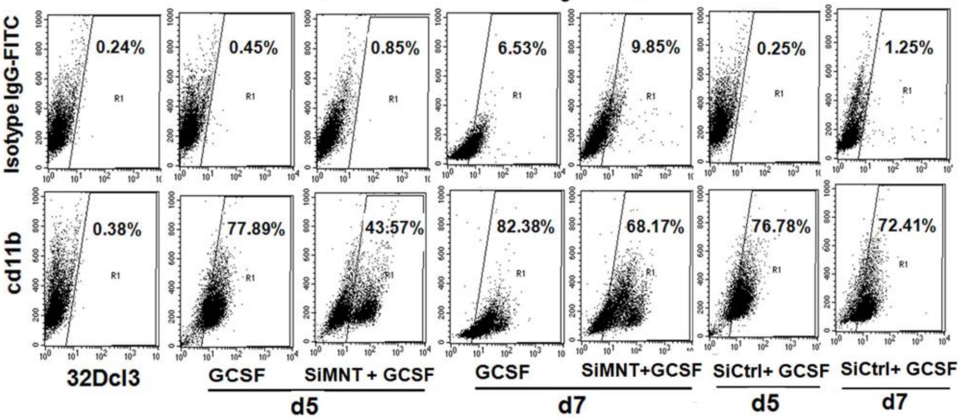

**S6: (a)** Western blot analysis of nuclear/ cytoplasmic fractions of U937 cells transfected with siMNT (50nM) for 0, 24, 48 and 72h. **(b)** graphical representation of cd11b expression as assessed by FACS flow cytometry in U937 cells transfected with siMNT (50nM) and co-treated with 5nM PMA for 24, 48 and 72h transfection. **(c)** Representative 2D-dot plot for FACS analysis of cd11b expression in U937 cells transfected with siMNT (50nM) for 0, 24, 48 and 72h and co-treated with PMA for 0, 24, 48 and 72h. **(d)** graphical representation of cd11b expression as assessed by FACS flow cytometry in 32Dcl3 cells transfected with siMNT (50nM) and co-treated with 100ng/ml G-CSF for day 0, d5 and d7. **(e)** Representative 2D-dot plot for FACS analysis of cd11b expression in 32Dcl3 cells transfected with siMNT (50nM) and co-treated with 100ng/ml G-CSF for day 0, d5 and d7.

**S7.a**

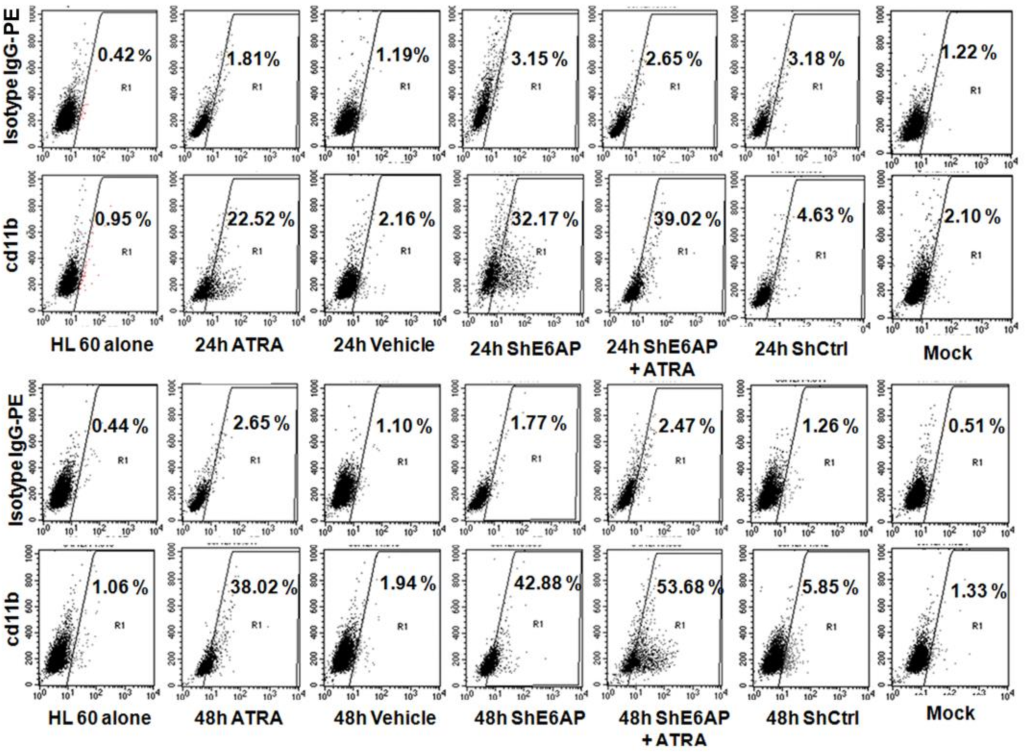


**S7.b**

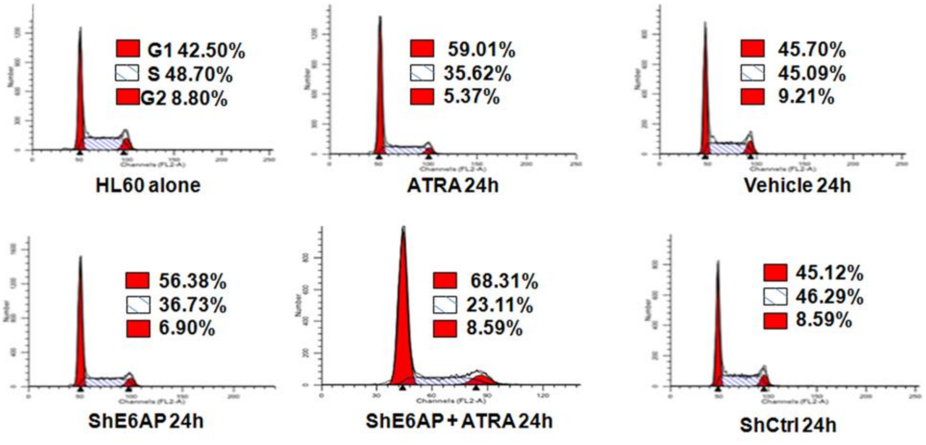

**S7: (a)** Representative 2D-dot plot for cd11b expression in HL60 cells transfected with shE6AP (1.0µg) and co-treated with ATRA for 0, 24 and 48h as assessed by FACS flow cytometry. **(b)** Representative cell cycle analysis of HL60 cells transfected with sh-E6AP (1.0 µg) and co-treated with ATRA for 0 and 24h in different phases of the cell cycle.

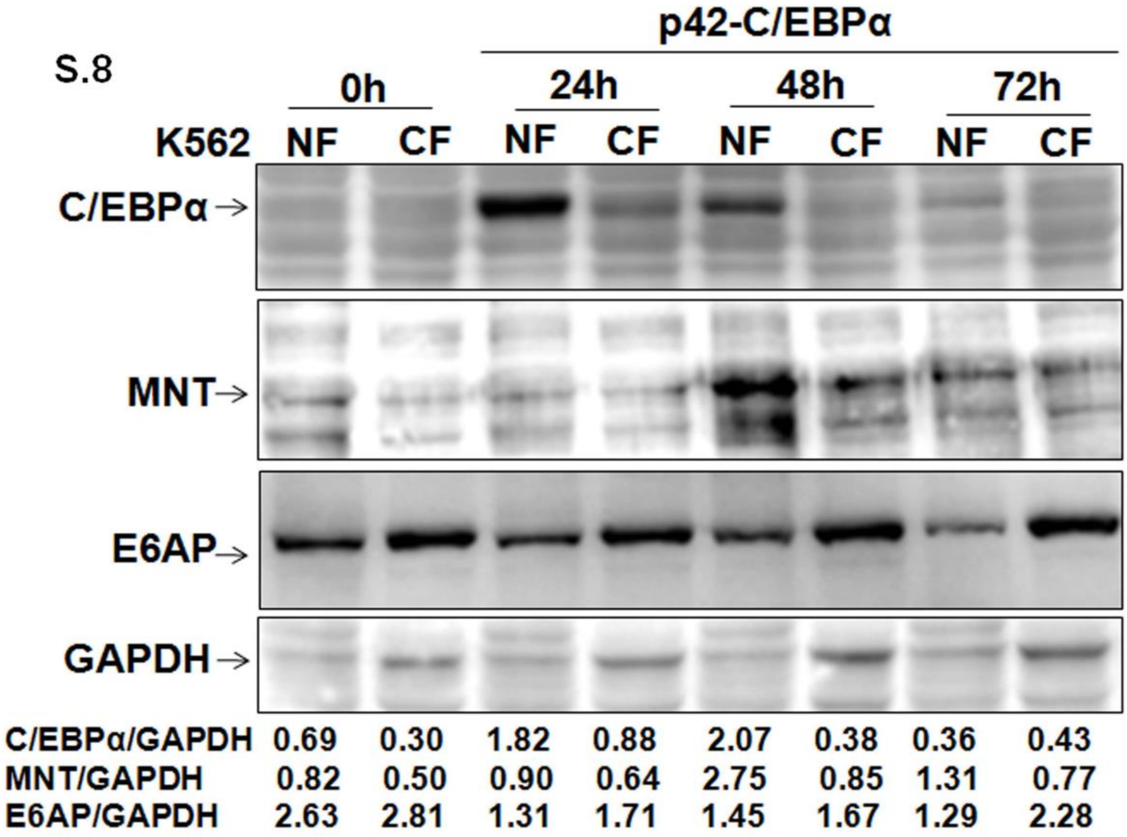


**S8: (a)** Ectopic expression of C/EBPα in K562 cells: C/EBPα (1.0µg) was transiently transfected in K562 cells. Post-transfection after indicated time points nuclear/ cytoplasmic fractions were prepared and immunoblotting was performed with anti-C/EBPα, anti-MNT and E6AP antibodies. GAPDH was used as loading control.

**Table S1**

| S. No. | Identified Protein | Accession No. | Score | Molecular Weight (Da) | pI | Function |
|---|---|---|---|---|---|---|
| 1. | Keratin, type II cytoskeletal I | P04264 | 44 | 66170 | 8.15 | May regulate the activity of kinases such as PKC and SRC via binding to integrin beta-1 (ITB1) and the receptor of activated protein kinase C (RACK1/GNB2L1 |
| 2. | Keratin type II cytoskeletal 6B | P04259 | 67 | 60315 | 8.09 | Structural constituent of cytoskeletal; ectoderm development |
| 3. | Protein disulfide-isomerase A3 | P30101 | 219 | 57146 | 5.98 | Catalyzes the rearrangement of -S-S- bonds in proteins |
| 4. | Keratin, type II cytoskeletal I | P04264 | 30 | 66170 | 8.15 | |
| 5. | Keratin, type II cytoskeletal I | P04264 | 30 | 66170 | 8.15 | |
| 6. | Not identified | | | | | |
| 7. | Not identified | | | | | |
| 8. | Keratin, type II cytoskeletal I | P04264 | 56 | 66170 | 8.15 | |
| 9. | Nil | | | | | |
| 10. | Actin-related protein 3 | P61158 | 635 | 47797 | 5.61 | Component of the NuA4 histone acetyltransferase complex which is involved in transcriptional activation of select genes principally by acetylation of nucleosomal histones H4 and H2A. This modification may both alter nucleosome – DNA interactions and promote interaction of the modified histones with other proteins which positively regulate transcription. This complex may be |

| | | | | | | |
|---|---|---|---|---|---|---|
| | | | | | | required for the activation of transcriptional programs associated with oncogene and proto-oncogene mediated growth induction, tumor suppressor mediated growth arrest and replicative senescence, apoptosis, and DNA repair. The NuA4 complex ATPase and helicase activities seem to be, at least in part, contributed by the association of RUVBL1 and RUVBL2 with EP400. NuA4 may also play a direct role in DNA repair when recruited to sites of DNA damage. |
| 11. | Glial Fibrillary acidic protein | P14136 | 70 | 49907 | 5.42 | GFAP, a class-III intermediate filament, is a cell-specific marker that, during the development of the central nervous system, distinguishes astrocytes from other glial cells. |
| 12. | Max- binding protein MNT | Q99583 | 42 | 62318 | 8.78 | Negative regulation of cell proliferation, transcriptional corepressor activity, regulation of cell-cycle, negative regulation of apoptotic signalling pathway |
| 13. | Ubiquitin protein ligase E3A | Q05086 | 113 | 101593 | 5.12 | E3 ubiquitin-protein ligase accepts ubiquitin from an E2 ubiquitin-conjugating |

| | | | | | | |
|---|---|---|---|---|---|---|
| | | | | | | enzyme in the form of a thioester and transfers it to its substrates. Several substrates have been identified including the RAD23A and RAD23B, MCM7 (which is involved in DNA replication), annexin A1, the PML tumor suppressor, and the cell cycle regulator CDKN1B. Catalyzes the high-risk human papilloma virus E6-mediated ubiquitination of p53/TP53, contributing to the neoplastic progression of cells infected by these viruses. Additionally, may function as a cellular quality control ubiquitin ligase by helping the degradation of the cytoplasmic misfolded proteins. Finally, UBE3A also promotes its own degradation in vivo |
| 14. | Max- binding protein MNT | Q99583 | 39 | 62318 | 8.78 | Negative regulation of cell proliferation, transcriptional corepressor activity, regulation of cell-cycle, negative regulation of apoptotic signalling pathway |
| 15. | Protein disulfide-isomerase A6 | Q15084 | 586 | 48490 | 4.95 | May function as a chaperone that inhibits aggregation of misfolded proteins. Plays a role in platelet aggregation and activation by agonists such as convulxin, collagen and |

| | | | | | | |
|---|---|---|---|---|---|---|
| | | | | | | thrombin. |
| 16. | Keratin type II Cytoskeletal I | P04264 | 45 | 66170 | 8.15 | |
| 17. | Ubiquitin-protein ligase E3A | Q05086 | 48 | 101593 | 5.12 | |
| 18. | Keratin type II cytoskeletal I | P094264 | 30 | 66170 | 8.15 | |
| 19. | Ubiquitin-protein ligase E3A | Q05086 | 37 | 101593 | 5.12 | |
| 20. | Keratin type II Cytoskeletal I | P094264 | 66 | 66170 | 8.15 | |
| 21. | Nil | | | | | |
| 22. | Calreticulin | P27797 | 379 | 48283 | 4.29 | Calcium-binding chaperone that promotes folding, oligomeric assembly and quality control in the endoplasmic reticulum (ER) via the calreticulin/calnexin cycle. This lectin interacts transiently with almost all of the monoglucosylated glycoproteins that are synthesized in the ER. Interacts with the DNA-binding domain of NR3C1 and mediates its nuclear export. |
| 23. | Not identifed | | | | | |
| 24. | Calreticulin | P27797 | 410 | 48283 | | |
| 25. | Nil | | | | | |
| 26. | Putative elongation factor 1-alpha-like-3 | Q5VTE0 | 35 | 50495 | 9.15 | This protein promotes the GTP-dependent binding of aminoacyl-tRNA to the A-site of ribosomes during protein biosynthesis |
| 27. | Ubiquitin-protein ligase E3A | Q05086 | 62 | 101593 | 5.12 | |
| 28. | Keratin type I cytoskeletal 9 | P35527 | 37 | 62255 | 5.14 | May serve an important special function either in the mature palmar and plantar skin tissue or in the morphogenetic program of the formation of these tissues. Plays a role in keratin filament |

| | | | | | | |
|---|---|---|---|---|---|---|
| | | | | | | assembly |
| 29. | Keratin type II cytoskeletal I | P04264 | 85 | 66170 | 8.15 | |
| 30. | Glutamate [NMDA] receptor subunit epsilon- 1 | Q12789 | 31 | 166489 | 6.67 | Required for RNA polymerase III-mediated transcription. Component of TFIIIC that initiates transcription complex assembly on tRNA and is required for transcription of 5S rRNA and other stable nuclear and cytoplasmic RNAs. Binds to the box B promoter element |
| 31. | Not identified | | | | | |
| 32. | Ubiquitin-protein ligase E3A | Q05086 | 50 | 101593 | 5.12 | |
| 33. | Glutamate [NMDA] receptor subunit epsilon- 1 | Q12789 | 37 | 166489 | 6.67 | |
| 34. | Not identified | | | | | |
| 35. | Not identified | | | | | |
| 36. | Glutamate [NMDA] receptor subunit epsilon- 1 | Q12789 | 37 | 166489 | 6.67 | |
| 37. | Nil | | | | | |
| 38. | Keratin, type II cytoskeletal 4 | P19013 | 40 | 57649 | 6.25 | |
| 39. | Protein disulfide-isomerase A6 | Q15084 | 586 | 48490 | 4.95 | May function as a chaperone that inhibits aggregation of misfolded proteins. Plays a role in platelet aggregation and activation by agonists such as convulxin, collagen and thrombin. |
| 40. | Glucose-regulated protein | P11021 | 69 | 72288 | 5.07 | Probably plays a role in facilitating the assembly of multimeric protein complexes inside the endoplasmic reticulum. Involved in the correct folding of proteins and degradation of misfolded proteins via its interaction with |

| | | | | | | |
|---|---|---|---|---|---|---|
| | | | | | | DNAJC10, probably to facilitate the release of DNAJC10 from its substrate |
| 41. | Nil | | | | | |
| 42. | Nuclear protein MDM1 | QD9067 | 56 | 79641 | 9.51 | Retina development in camera-type eye |
| 43. | Actin, cytoplasmic 1 | P60709 | 103 | 41710 | 5.29 | Actins are highly conserved proteins that are involved in various types of cell motility and are ubiquitously expressed in all eukaryotic cells |
| 44. | Beta-actin-like-protein 2 | Q562R1 | 103 | 41976 | 5.39 | |
| 45. | Actin, cytoplasmic 1 | P60709 | 103 | 41710 | 5.29 | |
| 46. | Transaldolase | P37837 | 374 | 37688 | 6.36 | Part a large chaperone multiprotein complex comprising DNAJB11, HSP90B1, HSPA5, HYOU, PDIA2, PDIA4, PDIA6, PPIB, SDF2L1, UGT1A1 and very small amounts of ERP29, but not, or at very low levels, CALR nor CANX. Interacts with MICA on the surface of tumor cells, leading to MICA disulfide bond reduction which is required for its release from tumor cells. Interacts with ITGB3 following platelet stimulation. |
| 47. | Beta-actin-like-protein 2 | Q562R1 | 78 | 41976 | 5.39 | |
| 48. | Actin, aortic smooth muscle | P62737 | 42 | 41982 | 5.23 | |
| 49. | Heat shock protein HSP 90-beta | P11499 | 76 | 83273 | 4.97 | Molecular chaperone that promotes the maturation, structural maintenance and proper regulation of specific target proteins involved for instance in cell cycle |

| | | | | | | |
|---|---|---|---|---|---|---|
| | | | | | | control and signal transduction. Undergoes a functional cycle that is linked to its ATPase activity. This cycle probably induces conformational changes in the client proteins, thereby causing their activation. Interacts dynamically with various co-chaperones that modulate its substrate recognition, ATPase cycle and chaperone function |
| 50. | Nil | | | | | |
| 51. | Nil | | | | | |
| 52. | 40S ribosomal protein S15a | P62245 | 41 | 14830 | 10.14 | Structural constituent of ribosome, positive regulation of cell cycle, ribonucleoprotein; ribosomal protein |
| 53. | Nil | | | | | |
| 54. | Nil | | | | | |
| 55. | Serine protease inhibitor Kazal-type 11 | Q09TK7 | 40 | 10083 | 7.55 | Probable serine protease inhibitor |
| 56. | Keratin, type II cytoskeletal I | P04264 | 30 | 66170 | 8.15 | |
| 57. | Glutamate [NMDA] receptor subunit epsilon- 1 | Q12789 | 37 | 166489 | 6.67 | |
| 58. | Nil | | | | | |
| 59. | Rho GDP-dissociation inhibitor 2 | P52566 | 205 | 23031 | 5.10 | Regulates the GDP/GTP exchange reaction of the Rho proteins by inhibiting the dissociation of GDP from them, and the subsequent binding of GTP to them. |
| 60. | Nil | | | | | |
| 61. | Nil | | | | | |
| 62. | Nil | | | | | |
| 63. | Calmodulin | P62158 | 120 | 16827 | 4.09 | Calmodulin mediates the control of a large number of enzymes, |

| | | | | | | |
|---|---|---|---|---|---|---|
| | | | | | | ion channels and other proteins by $Ca^{2+}$. Among the enzymes to be stimulated by the calmodulin-$Ca^{2+}$complex are a number of protein kinases and phosphatases. Together with CEP110 and centrin, is involved in a genetic pathway that regulates the centrosome cycle and progression through cytokinesis. |
| 64. | Nil | | | | | |

* MOWSE score of all the proteins are greater than the Mascot significant score. Accession number in UniProtKB/Swiss-Prot database.

The peptide mass data were analyzed for corresponding protein matching in the Swiss-Prot database with settings of Peptide Mass Tolerance: ± 150 ppm, Fragment Mass Tolerance: ± 0.3 Da and Max Missed Cleavages: 1 in MS/MS ion search using MASCOT search engine. Positive identification was considered only with a MASCOT score greater than Mascot significant score (35).
Peptide Mass Tolerance: ± 150 ppm
Fragment Mass Tolerance: ± 0.3 Da
Max Missed Cleavages: 1

**Fig. S9 (i): Mascot search result of Spot No. 12 based on MS and MS/MS analysis**

# Mascot Search Results

| | |
|---|---|
| **User** | : |
| **Email** | : |
| **Search title** | : |
| **Database** | : **UniProtKB-SwissProt sprot_20100615 (517802 sequences; 182492287 re** |
| **Taxonomy** | : **Homo sapiens (human) (20355 sequences)** |
| **Timestamp** | : **13 Jun 2011 at 09:18:59 GMT** |
| **Protein hits** | : Q99583 Max-binding protein MNT OS=Homo sapiens GN=MNT PE=1 SV=1 |
| | P04264 Keratin, type II cytoskeletal 1 OS=Homo sapiens GN=KRT1 PE= |
| | Q12879 Glutamate [NMDA] receptor subunit epsilon-1 OS=Homo sapiens |
| | Q05086 Ubiquitin-protein ligase E3A OS=Homo sapiens GN=UBE3A PE=1 |
| | P01562 Interferon alpha-1/13 OS=Homo sapiens GN=IFNA1 PE=1 SV=1 |

## Mascot Score Histogram

Ions score is -10*Log(P), where P is the probability that the observed match is a random event.
Individual ions scores > 29 indicate identity or extensive homology (p<0.05).
Protein scores are derived from ions scores as a non-probabilistic basis for ranking protein hits.

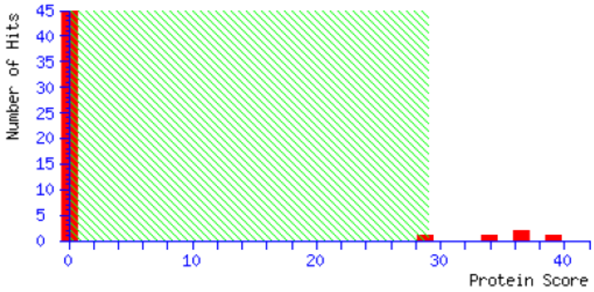


**Fig.S9 (i):** Mascot database search based on PMF and MS/MS matched to MNT with significant mowse score.

## Fig.S9 (ii) Percentage coverage of matched peptides for MNT

# Mascot Search Results

## Protein View

Match to: **Q99583** Score: **42**
**Max-binding protein MNT OS=Homo sapiens GN=MNT PE=1 SV=1**

Nominal mass ($M_r$): **62318;** Calculated pI value: **8.78**
NCBI BLAST search of Q99583 against nr
Unformatted sequence string for pasting into other applications

Taxonomy: Homo sapiens

Fixed modifications: Carbamidomethyl (C)
Variable modifications: Oxidation (M)
Cleavage by Trypsin: cuts C-term side of KR unless next residue is P
Sequence Coverage: **1%**

Matched peptides shown in **Bold Red**

```
  1 MSIETLLEAA RFLEWQAQQQ QRAREEQERL RLEQEREQEQ KKANSLARLA
 51 HTLPVEEPRM EAPPLPLSPP APPPAPPPPL ATPAPLTVIP IPVVTNSPQP
101 LPPPPPLPAA AQPLPLAPRQ PALVGAPGLS IKEPAPLPSR PQVPTPAPLL
151 PDSKATIPPN GSPKPLQPLP TPVLTIAPHP GVQPQLAPQQ PPPPTLGTLK
201 LAPAEEVKSS EQKKRPGGIG TREVHNKLEK NRRAHLKECF ETLKRNIPNV
251 DDKKTSNLSV LRTALRYIQS LKRKEKEYEH EMERLAREKI ATQQRLAELK
301 HELSQWMDVL EIDRVLRQTG QPEDDQASTS TASEGEDNID EDMEEDRAGL
351 GPPKLSHRPQ PELLKSTLPP PSTTPAPLPP HPHPHPHSVA LPPAHLPVQQ
401 QQPQQKTPLP APPPPPAAPA QTLVPAPAHL VATAGGGSTV IAHTATTHAS
451 VIQTVNHVLQ GPGGKHIAHI APSAPSPAVQ LAPATPPIGH ITVHPATLNH
501 VAHLGSQLPL YPQPVAVSHI AHTLSHQQVN GTAGLGPPAT VMAKPAVGAQ
551 VVHHPQLVGQ TVLNPVTMVT MPSFPVSTLK LA
```

**Fig.S9 (ii).** Mascot search result: Protein view showing matched peptides in bold red

**Fig. S9 (iii) PMF of MS analysis of Spot No. 12**

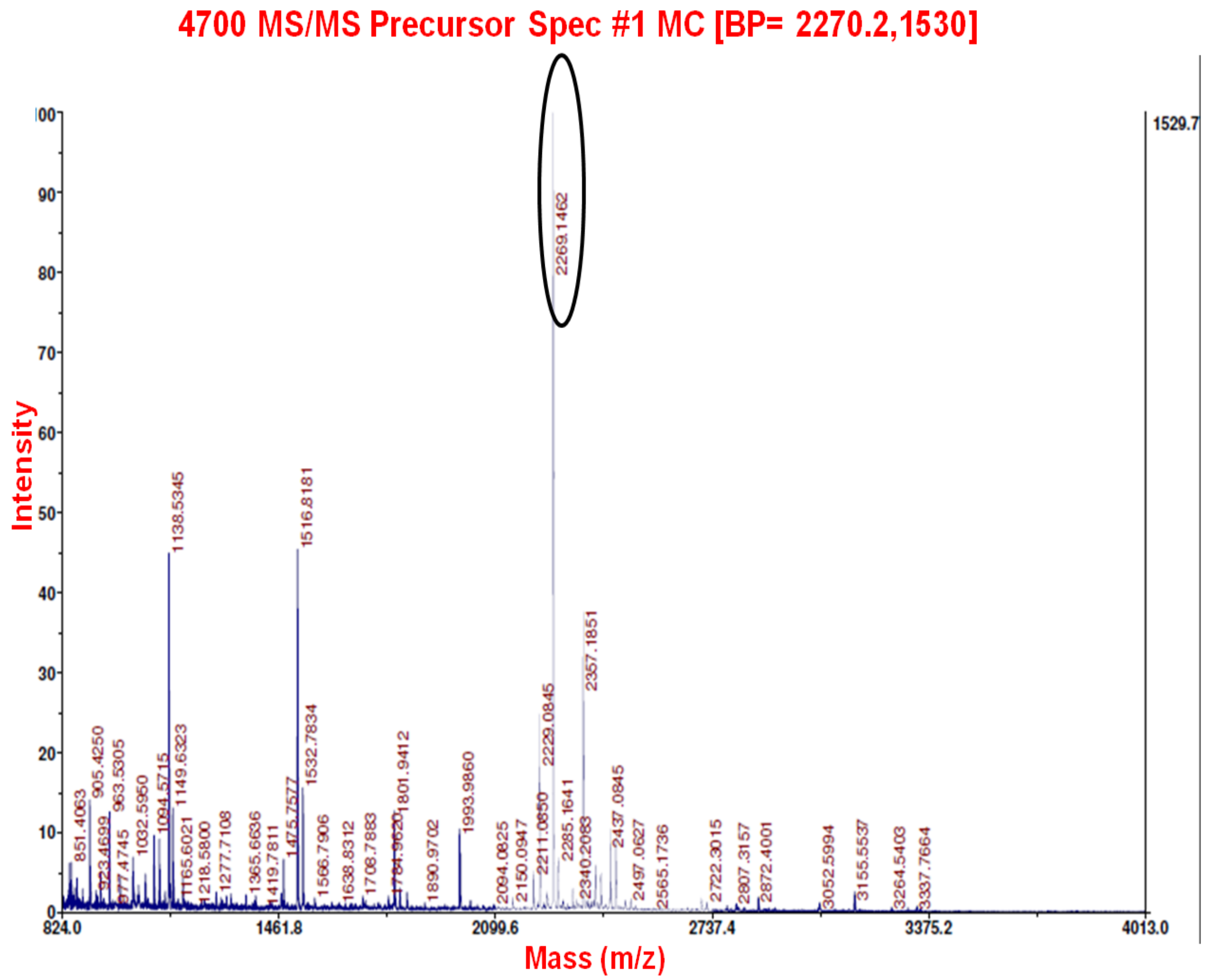


**Fig.S9 (iii).** Peptide mass fingerprint (PMF) generated from processed tryptic peptides from spot no. 12 and highest ion score peak was circled.

**Fig.S9 (iv) MS/MS of selected precursor ion with mass 2269.15 from PMF of spot No.12**

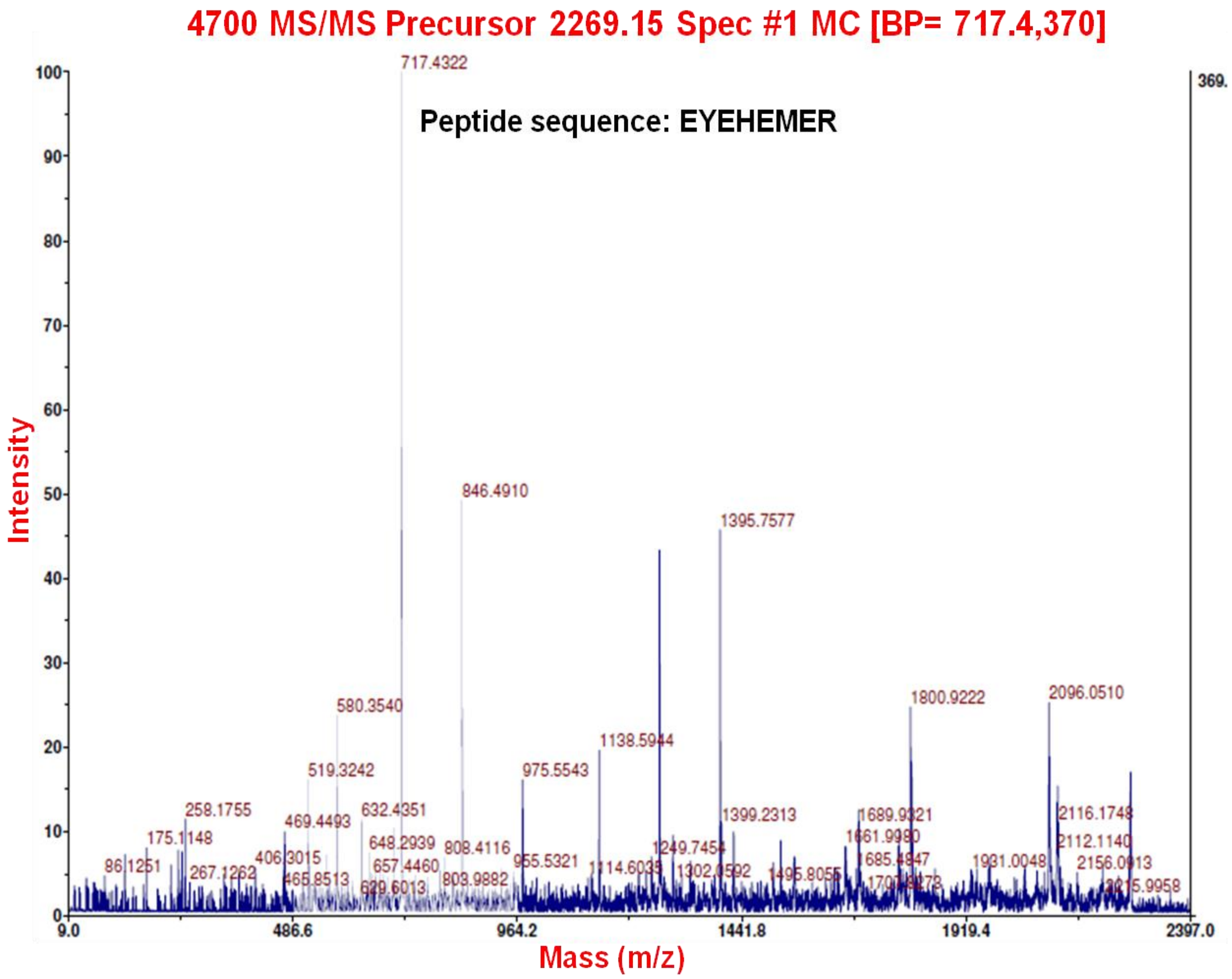


**Fig.S9 (iv):** MS/MS peak of highest ion score precursor peptide of spot No. 12
